# Three-Dimensional Spheroids as In Vitro Preclinical Models for Cancer Research

**DOI:** 10.3390/pharmaceutics12121186

**Published:** 2020-12-06

**Authors:** Bárbara Pinto, Ana C. Henriques, Patrícia M. A. Silva, Hassan Bousbaa

**Affiliations:** 1 Cooperativa de Ensino Superior Politécnico e Universitário (CESPU), Instituto de Investigação e Formação Avançada em Ciências e Tecnologias da Saúde (IINFACTS), Instituto Universitário de Ciências da Saúde (IUCS), 4585-116 Gandra PRD, Portugal; barbara_fernandes_pinto@hotmail.com (B.P.); A24955@alunos.cespu.pt (A.C.H.); patricia.silva@cespu.pt (P.M.A.S.); 2 Instituto Nacional de Engenharia Biomédica (INEB), Universidade do Porto, 4099-002 Porto, Portugal

**Keywords:** 3D cultures, tumor microenvironment, tumor spheroids, efficacy analysis, drug resistance, cancer therapy

## Abstract

Most cancer biologists still rely on conventional two-dimensional (2D) monolayer culture techniques to test in vitro anti-tumor drugs prior to in vivo testing. However, the vast majority of promising preclinical drugs have no or weak efficacy in real patients with tumors, thereby delaying the discovery of successful therapeutics. This is because 2D culture lacks cell–cell contacts and natural tumor microenvironment, important in tumor signaling and drug response, thereby resulting in a reduced malignant phenotype compared to the real tumor. In this sense, three-dimensional (3D) cultures of cancer cells that better recapitulate in vivo cell environments emerged as scientifically accurate and low cost cancer models for preclinical screening and testing of new drug candidates before moving to expensive and time-consuming animal models. Here, we provide a comprehensive overview of 3D tumor systems and highlight the strategies for spheroid construction and evaluation tools of targeted therapies, focusing on their applicability in cancer research. Examples of the applicability of 3D culture for the evaluation of the therapeutic efficacy of nanomedicines are discussed.

## 1. Introduction

Significant investments are made in cancer research for drug discovery and development. Yet, the approval rate (≤5%) of drugs that reach the clinic remains very low [1,2]. Typically, anticancer compounds are tested in two dimensional (2D) cell culture models, that involve a panel of cancer cell lines, such as those used by the US National Cancer Institute [3]. Drugs that show promising cytotoxicity in 2D in vitro system progress to animal models of human cancers (mainly mice) for anti-tumor efficacy testing [4]. Unfortunately, most of the promising preclinical drugs have no or weak efficacy in real patients with tumors, resulting in a significant delay of anticancer drug development [5]. One of the main factors underlying this poor success is the inadequacy of the preclinical 2D cultures and animal models to recapitulate the human tumor microenvironment (TME). TME is a complex and heterogeneous structure made of cellular (e.g., transformed epithelial cells, fibroblasts, infiltrating lymphocytes, mesenchymal stem cells, endothelial cells) and non-cellular (e.g., extracellular matrix—ECM, growth factors, cytokines and chemokines) components, with a critical role in cancer development and progression [6,7]. The 2D culture systems lack the structural architecture and the microenvironment of the tumor, and display altered gene expression and activation of cell signaling pathways, compared to the in vivo tumor tissues (Table 1) [8,9,10]. Besides the associated higher cost and ethical issues, animal models also display significant limitations and poorly reflect the proprieties of human tumors. For instance, the stromal component of the xenograft is not of human origin, the rate of growth is higher in xenografts (doubling time of a few days) than in primary human tumors (doubling time of a few months), and, thus, they often tend to respond better to anticancer drugs [11].

Therefore, the development of preclinical models that better recapitulate patient tumor and microenvironment represents a promising challenge to improve the success rates in anticancer drug development. Since the discovery of the importance of the extracellular matrix (ECM) in cell behavior, it became clear that three-dimensional (3D) cell culture systems offer an excellent opportunity to recapitulate the real avascular tumor, by allowing cancer cells to be cultured, either alone or in co-culture with other cell types, in a spatial manner reminiscent of the structural architecture of the tumor that provides cell–cell and cell–ECM interactions, thereby mimicking the native tumor microenvironment (Table 1) [12,13,14,15]. Hopefully, besides circumventing the barriers and limitations imposed by 2D monolayer cultures, 3D cell culture models could reduce or, ideally, replace the use of animal models, thereby resolving the associated ethical and cost issues [16,17]. Here, common 3D cell culture methods are highlighted, the characterization tools for the evaluation of the targeted effect are reviewed, with focus on multicellular tumor spheroids (MCTS) and their applicability in cancer research.

## 2. Tumor Microenvironment as Pathophysiologic Barrier to Anticancer Therapy

**Figure 1:**
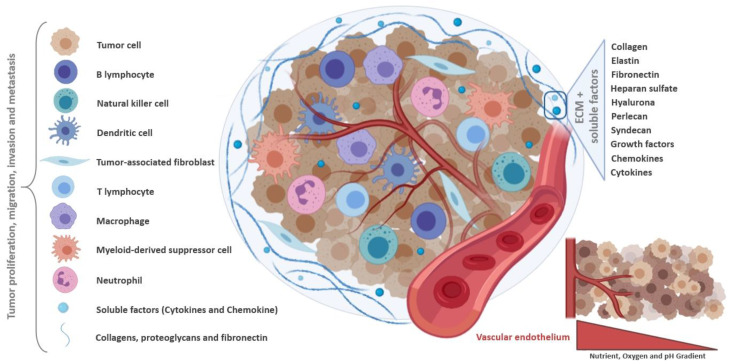
Schematic representation of the tumor microenvironment. The tumor ecosystem consists of a heterogeneous population formed by cancer and infiltrating immune cells, including tumor-associated fibroblasts, myeloid-derived suppressor cells and immune cells. The cross-talk between all these tumor microenvironment components play an essential role in tumor growth, development and metastasis, under hostile conditions. Soluble factors are constantly produced, triggering immunosuppressive responses and tumor survival. Created with BioRender.com.

The TME comprises the heterogeneous population of malignant cells, the ECM, and various tumor-associated cells such as cancer-associated fibroblasts (CAF), endothelial cells, adipocytes, and immune cells (Figure 1). Tumor-associated macrophages (TAMs) are monocyte-derived macrophages that can be categorized as inflammatory M1 macrophages, with roles in phagocytosis and cell killing, and immunosuppressive M2 macrophages, with roles in tissue repair [18]. The TME, mainly through hypoxia and secreted cytokines, promotes the M2 phenotype which favors, amongst others, tissue repair and tumor invasion and progression [19,20]. TAMs can constitute up to 50% of the tumor mass, and are associated with poor prognosis in many cancer types. CAFs are also a major component of the TME, characterized by a high interaction with tumor cells and the TME. In this context, CAFs contribute to tumor cell invasion, as well as to changes in tumor growth and immune microenvironment, through ECM remodeling and production of soluble factors [21,22].

The ECM provides structural support for cells in the extracellular space, and is composed of structural fibrous proteins (e.g., collagens and elastin), multiadhesive proteins (e.g., fibronectin and laminin), glycosaminoglycans (e.g., heparan sulfate, hyaluronan), proteoglycans (e.g., perlecan, syndecan), and sequestered growth factors, as well as secreted proteins [23,24,25]. The cross-talk between the different TME cells and components plays an essential role in tumor growth, progression and metastasis [26].

Many factors present in the TME, including transforming growth factor beta (TGF-β), cytokines (IL-10 and IL-1β), members of the VEGF, plateled-derived growth factor (PDGF), FGF, angiopoietin families, Bv8/PROK2, and hypoxia-inducible factor (HIF)-1α, provide molecular support to tumor growth and progression [27,28,29,30]. Additionally, cancer cells are experts in modifying their surrounding environments. For instance, cancer cells can co-opt fibroblasts to obtain growth factors, such as basic fibroblast growth factor (bFGF), necessary to sustain their growth and proliferation. additionally, tumor cells can interact with the surrounding endothelial cells, promoting the release of soluble factors, like vascular endothelial growth factor (VEGF), to trigger the angiogenic process. Tumor cells can also evade the immune-mediated cellular destruction through different strategies. For instance, loss of tumor antigen expression precludes their recognition by the immune system, production of immunosuppressive cytokines protects them from the cytotoxic lysis by immune cells, and development of immunosuppressive forces leads to local immunosuppression in the TME that shifts the phenotype and function of normal immune cells from an anti-tumor state to a pro-tumor state [31,32,33,34,35,36].

Currently, treatment options against cancer include surgery, chemotherapy, radiation therapy, hormonal therapy, and targeted therapy [37]. Basically, anticancer therapies aim to target tumor cells either directly, through DNA damage by cytotoxic drugs or local radiation causing apoptosis, or indirectly, through the destruction of TME so as to deprive cancer cells of the machinery that fuel their growth and progression. However, these therapies induce new biological tumor responses, mainly through immunological and angiogenic modulation, contributing to drug resistance, which remains a serious consequence of most anticancer treatments, impacting the patient’s prognosis and quality of life [31,38].

The TME imposes many biological barriers that greatly hinder drug delivery to tumors [39,40]. These barriers include malformed vasculature, rigid extracellular matrix, hypoxia, acidic pH, abnormal enzyme level, altered metabolism pathway, and immunosuppressive environment. Uncontrolled cell growth and proliferation result in insufficient blood supply to cancer cells in the inner core and in the intermediate layer of the tumor mass, causing cellular hypoxia [39]. Hypoxia, one of the hallmarks of cancer, plays a fundamental role in tumor development and malignancy. This condition is able to modify the tumor endothelial cells morphology, reducing oxygen diffusion to cancer tissue [41]. While hypoxia is harmful to non-tumor cells, unfortunately, cancer cells readily switch from oxidative phosphorylation to aerobic glycolysis, a condition known as Warburg effect, orchestrated by the transcription factor HIF-1α through which cancer cells acquire many malignant properties [42,43]. Moreover, the tumor vessels exhibit a disordered structure, which leads to a decrease in the blood perfusion homogeneity [44]. This tumor vascular deficiency makes difficult drug distribution to all cancer cells, impacting therapy effectiveness [43]. TME pH also contributes to anticancer drug resistance. The increase in anaerobic metabolism leads to greater lactic acid production, reducing the extracellular pH, that ranges from 6.2 to 7.2 [45]. As pH levels decrease, metalloproteinases become activated, destroying cell interactions which facilitates tumor migration and invasion [46]. Acidic microenvironment causes the “ion trapping” phenomenon, process in which basic anticancer drugs are transformed into a cation substance, reducing their transmembrane permeability and, consequently, their effectiveness [47]. Immune cells such as macrophages, neutrophils, mast cells, myeloid-derived suppressor cells, and natural killer cells, secret many soluble factors that promote immunosuppression, angiogenesis, chronic inflammation, and drug resistance [43,48,49,50]. Additionally, the mechanisms associated with immune escape during tumor progression can promote resistance to anticancer drugs [31,43]. Tumor cells themselves can alter the organization and protein deposition of the ECM, forming a physical barrier that prevents drug penetration into tumor cells [51,52].

Therefore, new therapeutic strategies have been developed to target the tumor-promoting microenvironmental factors in a goal to block the interaction between tumor cells and the TME [53]. Such strategies include, for example, inhibition of the extracellular ligand-receptor interactions and downstream pathways, re-programming the immune response, and co-targeting of tumor cells and the microenvironment [43].

As outlined above, the tumors and their microenvironment provide multiple biological barriers against drug penetration, accumulation, and efficacy, leading to tumor resistance to therapy [54]. Thus, discovery and delivery testing of new anticancer drug candidates require preclinical models that are more physiological than conventional 2D cultures, capable of recapitulating these TME barriers. In this sense, the spheroids provide the appropriate model of the pathophysiologic parameters present in the real tumor, because they recapitulate the complex multicellular architecture, the barriers to mass transport, and extracellular matrix deposition, which explain their growing use as models for better prediction of drug effects and delivery in the last decades [55].

## 3. Common Characteristics of Spheroids and Tumors

**Figure 2:**
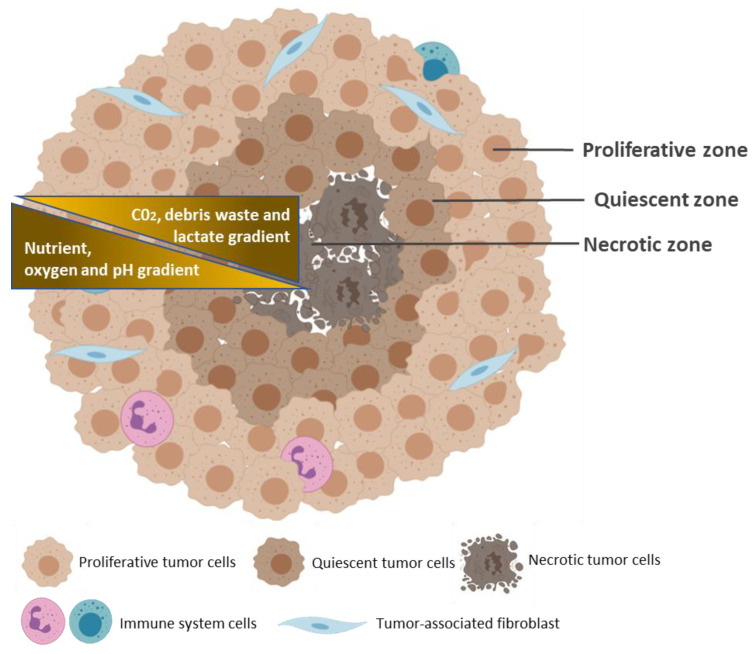
Typical structure of a multicellular tumor spheroid. The geometric rearrangement of the cells within the spheroid forms three concentric zones of heterogeneous cell populations: an external proliferating zone (Proliferative zone); a middle zone of quiescent cells (Quiescent zone), and an internal zone of necrotic cells (Necrotic zone). These cell layers are caused by the gradients of nutrients, oxygen, and pH (yellow), from the outside to the center of the spheroid, and by the gradients of CO_2_, waste, and lactate, from the center to the outside. Created with BioRender.com.

Various cancer cells can spontaneously assemble into spheroids in culture environment that privileges cell–cell and cell–ECM interactions over cell–substrate interactions [14]. These predominant cell–cell and cell–ECM interactions result in the formation of a 3D structure that closely reproduces mimics the native spatial organization and environment of avascular tumors, where cells can proliferate, aggregate and differentiate (Figure 2) [56]. Common methods for spheroid generation are described in the next section. Spheroids have a diameter of 200 micrometers or more, generally with a spherical shape, and display three concentric zones of heterogeneous cell populations: an external zone of highly proliferating and migrating cells; a middle zone of quiescent cells, and an internal zone of necrotic cells [57,58]. These cell layers are so defined due to the nutrients and oxygen gradients that are established, as a result of limited diffusion, from the outside to the center of the spheroids. Thus, cells of the peripheral layer of the spheroids are exposed to sufficient oxygen and growth factors from the medium, which stimulate their proliferation. At the middle layer, limited diffusion of growth factors forces cell entry into quiescent state of the cell cycle. In large spheroids (>500 micrometers), oxygen deficiency (hypoxia) in the innermost zone induces altered gene expression, through stabilization of the transcription factor HIF-1α, and, consequently, triggers the Warburg effect, promoting aerobic glycolysis and lactic acid production, thereby lowering pH of the inner layer of spheroids [59]. Nutrient and oxygen deprivation, together with the accumulation of metabolic waste, triggers the necrotic death of cells at the innermost layer.

Therefore, 3D culture systems recapitulate many characteristics of in vivo tumors, such as cell–cell and cell–ECM interactions, nutrient and oxygen gradients, and distinct layers of cell populations. Besides, the morphology and polarity of the cells, as well as gene expression and activation of cell signaling pathways, are also close to those of real tumors [8,9,60,61]. These features make spheroids a promising model for the study of cancer biology, cancer initiation, invasion and metastatic processes, as well as drug testing.

## 4. Methods for Spheroid Generation

Cells grown in culture environment of low binding or absence of adhesive surface can assemble into 3D spheroids, as these conditions favor cell–cell and cell–ECM interactions over cell–substrate interactions. Spheroids can be obtained after 1 to 7 days of culture, with various morphologies, depending on the cell line and the approach used. Examples of studies that performed spheroid generation techniques are shown in Table 2, providing information on cell lines, density and the time required to obtain the spheroids. According to the literature, spheroids of 300–500 µm of size are those that best mimic in vivo tumors in terms of hypoxia and proliferation gradients. Typically, spheroids are constructed from tumor cells, using scaffold-free or scaffold-based techniques [62].

### 4.1. Scaffold-Free Techniques

In scaffold-free techniques, different factors (e.g., low-adhesion substrates, gravity force and magnetic action) contribute to cellular aggregate formation and spheroid generation. During this process, the ECM is originated through continuous deposit of proteins produced by spheroid cells [63]. The most common scaffold-free techniques currently used are ultra-low attachment plates, hanging drop, magnetic levitation and magnetic 3D printing. The advantages and disadvantages of each method are summarized in Table 3.

#### 4.1.1. Ultra-Low Attachment Plates

The surfaces of the plates are coated with a substrate to prevent cell adhesion, promoting cell aggregation and spheroid formation. Besides presenting low adhesion, the wells of these plates have a defined shape (round bottom, V-shaped or conical), allowing the positioning of a single spheroid [64,65]. Generally, the main substrates used to coat the plate are agar/agarose or poly(2-hydroxyethyl methacrylate), by adding 50 µL of solution in each well of 96-well plates, at concentrations of 15 mg/mL and 5 mg/mL, respectively [66,67,68]. With this technique, a large number of spheroids can be generated simultaneously on the same plate, facilitating experimental reproducibility, in addition to enable the monitoring of spheroid formation and growth. As disadvantages, some tumor cell lines do not form tight spheroids in ultra-low attachment plates [69].

#### 4.1.2. Hanging Drop

In this method, approximately 25 µL of cell suspension is positioned inside of a petri dish lid, which contains phosphate-buffered saline (PBS) to avoid dehydration of the cellular solution. Then, the lid is inverted and due to surface tension, the droplets remain suspended. Owing to the force of gravity, the cells within the droplets spontaneously form cellular aggregates, giving rise to a single spheroid [70,71]. This method allows large production of spheroids and an easy control of their size. However, it is labor intensive due to its multistep process, and there is a risk of cell damage in case of media evaporation, requiring constant monitoring of the culture medium [71,72,73,74]. Moreover, the hanging drop method can originate spheroids with heterogeneous sizes and morphologies, impacting the spheroid standardization, which is essential for new drug screening. Recently, studies have developed different tools to minimize these limitations and facilitated the realization of this method [75,76,77]. For instance, the pressure-assisted network for droplet accumulation (PANDA) system consists of a pressure chip capable to create homogeneous and compact hanging drop array, enabling the fast and economical production of spheroids [75]. Another way to circumvent these barriers is through the use of 3D printed hanging-drop dripper array that allows in situ analysis of drug screening, tumor metastasis and tumor transendothelial migration, besides promoting heterotypic spheroid interaction [77].

#### 4.1.3. Magnetic Levitation and Magnetic 3D Printing

Through a mixture of magnetic particles/nanoparticles, the cells are magnetized and incubated under magnetic forces to overcome the gravitational force, allowing their levitation and, consequently, formation of cellular aggregate [78]. In this method, after the cells absorb the magnetic particles, a magnet is positioned above (magnetic levitation) or below (magnetic 3D printing) the plate, promoting cells aggregation and spheroid generation [70]. Spheroids are usually formed in less than 16 h, being considered a fast-acting technique [72]. However, prior preparation of magnetic nanoparticles is necessary, and limited number of spheroids are generated [78].

### 4.2. Scaffold-Based Techniques

In scaffold-based techniques, external cell anchoring systems are used to mimic the ECM structure, allowing greater cell–cell and cell–matrix interaction. These systems can consist of porous microcarriers of natural, synthetic, and semisynthetic hydrogels made of cross-linked polymers. Porous scaffolds are widely used in the bioengineering sector, and have gained prominence in 3D cell culture implementation [63,72,79]. Due to their interconnected pores, these structures allow greater diffusion of nutrients, oxygen and metabolic debris, in addition to mimicking the ECM architecture, providing cellular support, attachment and proliferation [80,81,82]. 3D porous scaffolds also promote the formation of bigger spheroids, compared to non-porous scaffolds, and enhance tumor cell invasion and therapeutic resistance [83,84]. Although they are synthesized mainly by polymers such as poly(ε-caprolactone), porous microparticles can consist of different substances, including natural (e.g., chitosan, hyaluronic acid, alginate, collagen, gelatin, silk fibroin), and synthetic (e.g., poly(lactide-co-glycolide)) materials [85,86,87,88,89,90]. The porosity and pore size of the scaffold are essential for the establishment of effective 3D models, as they can affect the transport of oxygen, metabolites and nutrients, as well as cell adhesion and cell growth [91]. While porous 3D scaffold methods are useful to control the spheroid size, effective collecting and separation of spheroids from 3D scaffolds may be difficult [92]. Common scaffold-based methodologies include spinner flasks, micropatterned plates, matrix encapsulation, matrix on top, matrix embedded, microcarriers beads, and microfluidic devices.

#### 4.2.1. Spinner Flasks

Continuous rotating agitation inhibits cell adhesion to the surface, leading to spheroid formation. The main means of rotation used are through spinner flasks and rotating flasks. In spinner flasks, a magnetic stirrer is positioned inside the flask, allowing homogeneous distribution of oxygen and nutrients. However, the cells are subjected to direct shearing force, which increases the risk of their damage. In the rotating flasks, the flask itself is rotated, allowing the dispersion of oxygen and nutrients, and the reduction of the shear forces on the cells [70]. As an advantage, this method allows large scale generation of spheroids. However, the continuous rotation prevents the visualization of the aggregates, can damage the cells, and is hard to monitor [71]. Yet, this method is considered as one of the most efficient systems for obtaining large amounts of spheroids under controlled nutritional conditions [72].

#### 4.2.2. Micropatterned Plates

The plates are modified to create micrometer sized compartments with a low adhesion surface within each microspace, providing a micropattern or microwells which induce cells to grow as clusters. First, a layer of 3-trimethoxysilyl polymethacrylate is added to the glass plate, to ensure fixation of the hydrogel microwells to the plate, followed by a uniform layer of hydrogel. Soon after, using photolithography techniques, polydimethylsiloxane is added to the hydrogel for microwell formation [70]. The cell suspension is then seeded into hydrogel microwells, which can vary in size from 150–600 µm [93]. This method allows large scale production of spheroids. However, bubbles often form during the culture, and pipetting can damage micropatterned surfaces due to pipetting [64].

#### 4.2.3. Matrix Encapsulation

Suspended cells are surrounded by hydrogel and placed in calcium free solution, forming cellular microcapsules. In these microcapsules, cells aggregate to form matrix encapsulated spheroids [70]. Generally, microcapsules have a size between 100 and 500 µm, are capable of generating monotypic or heterotypic spheroids, and allow cell–cell and cell–ECM interaction [94]. In these systems, the transport of nutrients and metabolic residues occurs by simple diffusion and, as the microcapsule increases, the nutrient transport becomes limited, which can cause cellular necrosis. Due to their viscoelastic capabilities, alginate hydrogels has been widely used to generate microcapsules [95]. An important advantage is that this method yields homogeneous sized spheroids.

#### 4.2.4. Matrix on Top and Matrix Embedded

The matrix on top and matrix embedded methods are quite similar. In the matrix-on-top method, the cells are seeded and trapped on the top of the solid matrix, and spheroids are formed through cellular aggregation. In the matrix embedded method, cells suspended in the liquefied matrix are only incorporated into the matrix after the gelation process [70]. Several compounds have been used as a matrix, including agarose, matrigel, collagen, and synthetic polymers [96]. Matrix-on-top method facilitates post-culture processing and imaging of the generated spheroids.

#### 4.2.5. Microcarrier Beads

This system has been used for more than 25 years for to generate 3D cell culture [97]. In this method, cells adhere to natural (e.g., collagen, cellulose) or synthetic (e.g., dextran, poly(d,l-lactide-co-glycolide)) matrix-coated beads, forming spheroidal structures [98,99]. The microcarrier beads provide a cell attachment surface, allowing the aggregation, especially of cells unable to aggregate spontaneously. This method is considered a fast, easy, and reproducible spheroid generation system, and allows the adhesion of different cell types to form heterogeneous spheroids. However, the presence of microcarrier beads in spheroids does not mimic the tumor physiological conditions in vivo [100].

#### 4.2.6. Microfluidic Devices

The cells are placed in microchannels with a free perfusion system, allowing the distribution of oxygen and nutrients, and the elimination of metabolic waste. As an advantage, this system can mimic tumor microvasculature in vivo. However, this method requires specialized laboratories and equipment [101,102,103]. Due to its ability to guarantee gases permeability, polydimethylsiloxane (PDMS) is the most used material for making microfluidic devices [104]. In addition, PDMS are biocompatible, easy to make, and are low cost. However, under high pressure, PDMS microchannels can be deformed, causing changes in fluid speeds. Depending on the type of sealing, reversible or irreversible, the PDMS microfluidic devices can withstand pressure up to 0.3 or 2 bar, respectively. Moreover, when exposed to some fluids, PDMS microfluidic can swell, which impacts in device function [105,106,107]. Other microfluidic device polymers, such as thermoset polyester, polyurethane methacrylate and Norland Adhesive 81, also undergo structural changes when exposed to pressures above 10, 8 and 5 bar, respectively [108].

**Table 2:** Examples of tumors and respective cell lines, densities and time required for 3D cell culture formation by different spheroid generation methods.

Spheroid Techniques	Spheroid Generation Methods	Tumor/Cell Lines	Cell Seeding Densities	Period to Spheroid Formation/Observations	References
**Scaffold-free techniques**	1. Ultra-low attachment plates	Head and neck squamous cell carcinoma lines (HNSCC): Cal33, Cal27, FaDu, UM-22B, BICR56, OSC-19, PCI-13, PCI52, Detroit-562, UM-SCC-1, and SCC-9.	625, 1250, 2500, 5 × 103, 1 × 104, or 2 × 104 cells/well.	Although some HNSCC cell lines formed MCTSs within 24 h of seeding into 384-well ULA-plates, others required 2–3 days to self-assemble.	[109]
		HNSCC cell lines: Cal33 and FaDu.	5 × 103 cells/well.	Typically, the spheroids were formed 24 h after seeding.	[110]
		Hepatocellular carcinoma cells: Huh-7; Hpatic stellate cells: LX-2.	Monospheroids: 750 cells/well of Huh-7 or 2250 cells/well of LX-2; Mixed-cell spheroids: Huh-7 and LX-2 cells/well at a 1:3 ratio (750:2250).	Spheroids were formed on day 1.	[111]
		Human bone marrow mesenchymal stem cells (hBM-MSCs).	1.4 × 104, 3.5 × 104, 1.4 × 104, and 3.5 × 104 cells/well.	Spheroids were observed 1 day after seeding; 96-well plates were pre-coated with 20 mg/mL of poly(2-hydroxyethyl methacrylate).	[112]
	2. Hanging drop	Human hepatoma cell line: HepG2.	1 × 104, 2 × 104, 4 × 104 and 5 × 104 cells/well.	Spheroid formation was observed 1 day after seeding.	[113]
		Human bone marrow mesenchymal stem cells: (hBM-MSCs).	1 × 104, 2.5 × 104, 1 × 105, 2.5 × 105 cells/droplet.	Spheroid formation was observed 1 day after seeding.	[112]
		Non-tumorigenic mammary cells: MCF10A; breast cancer cells: MDA-MB-231; and co-culture with MCF10A and mesenchymal stem/stromal cells (MSC).	MCF10A (3000 cells/droplet); MDA-MB-231(2000 cells/droplet); co-culture with MCF10A and MSC (cells were seeded at 1:1 with 2000 total cells/droplet).	Spheroid formation was observed 1 day after seeding.	[114]
		Murine colon carcinoma: CT26.	5000 cells/droplet.	Spheroid formation was observed 1 day after seeding.	[115]
**Scaffold-based techniques**	3. Magnetic levitation and Magnetic 3D printing	Human breast cancer cell line: MCF-7.	1000 cells/well.	Singular and concentrated 3D spheroids were observed 1 day after seeding; Cells suspended in a diethylenetriaminepentaacetic acid gadolinium (III) dihydrogen salt hydrate (Gd-DTPA) medium.	[116]
		Murine colon carcinoma: CT26 and human glioblastoma cells: U-87 MG.	1 × 106 cells/µL/mold.	Spheroid formation was observed 1 day after seeding; Magnetic spheroids of sizes of 0.4, 0.5, 1, and 1.6 mm were used.	[115]
		Human pancreatic β-cell line (EndoCbH3) and human umbilical vein endothelial cells (HUVECs).	5000 cells/50 mL in corresponding cell culture media per well; Cell ratio: 5000 cells to 5000 HUVECs.	The exact beginning of spheroid formation was not described; spheroid formation was observed from day 5; β-cells and HUVECs were previously treated with NanoShuttle™-PL at a concentration of 40 μL/mL in media culture.	[117]
		Mesenchymal stem cells (MSCs).	1 × 104 cells/well (before incubation with magnetic nanoparticles.	Spheroid formation was observed 1 day after seeding.	[118]
	4. Spinner flasks	Human hepatoma cell line: SK-Hep-1.	1 × 106 cells were inoculated into a siliconized Cellspin flask containing 250 mL of growth medium.	Cell aggregation was observed between 24–48 h after cell seeding. Spheroids were formed from 7–10 days, being well defined on day 10.	[119]
		Human adenocarcinoma cells: HT29.	5 × 104 HT29 cells per 75 cm2 flask; Aggregates were transferred to 250 mL spinner flasks containing 150 mL of culture medium.	Cell aggregation was observed from day 3 and spheroids were observed in day 15.	[120]
	5. Micropatterned plates	Sprague Dawley rats’ hepatocytes and MSC.	4 × 105 cells/well.	Spheroids formed gradually within 2 days.	[121]
	6. Matrix encapsulation	Mouse colon carcinoma cells: CT26.	3 × 106 cells/mL.	The exact beginning of spheroid formation was not described; spheroid formation was observed from day 5.	[122]
		Human umbilical vein endothelial cells (HUVECs) and mesenchymal stem cells (MSCs).	6 × 105 cells/mL (75% MSCs and 25% HUVECs).	Spheroid formation was observed 1 day after seeding.	[123]
	7. Matrix-on top and Matrix embedded	Neuroblastoma cells: SK-N-BE(2); lung cancer cells: H460; and glioblastoma cells: U87vIII.	4750 cells/ droplet.	Spheroids began to form 1 day after seeding.	[124]
		Human adenocarcinoma cell line derived from a metastatic site: MDA-MB 231 and murine Abelson leukemia transformed macrophage/monocyte line: RAW 264.7.	500 to 5000 of RAW 264.7 cells with 10,000 of MDA-MB 231 cells.	Spheroids began to form 1 day after seeding.	[125]
	8. Microcarriers beads	Human hepatocarcinoma cell line: BEL7402.	1 × 104 cells/well.	The exact beginning of spheroid formation was not described; on day 4 spheroids were already formed; It was added 200 microcarrier beads/well (Cytodex-3); The plate was coated with 10% poly(2-hydroxythyl methacrylate).	[126]
		Human melanoma cell line: BLM.	5 × 105 cells.	Spheroid formation was described on the first day.	[127]
	9. Microfluidic devices	Human cancer cell lines derived from ovarian solid tumor (TOV112D) or ascites (OV90).	12,000 cells/mL.	The exact beginning of spheroid formation was not described; spheroid formation was observed from day 3; Cell were seeded in single inlet multi-size spheroid synthesis (SIMSS) chips.	[128]
		Human colorectal cancer cell line: HT-29, and human normal fibroblast cell line: CCD-18Co.	5 × 106 /mL of HT-29 and 3 × 106 /mL of CCD-18Co.	Spheroid formation was observed 1 day after seeding; Microfluidic chips were made using PDMS.	[129]

**Table 3:** Advantages and disadvantages of the main methods used for spheroid generation.

Spheroid Techniques	Spheroids Generation Methods	Advantages	Disadvantages	References
**Scaffold-free techniques**	1. Ultra-low attachment plates 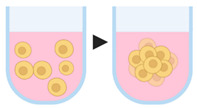	Large-scale spheroid production; Inexpensive; Easy handling.	Difficulty in forming tight spheroids in some cell lines.	[64,65,71,72,130,131]
	2. Hanging drop 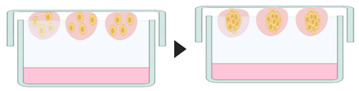	Production of up to 384 spheroids in a single trial; Control of cell composition and spheroid size; No specialized equipment or reagents required.	Difficulty in tracking spheroid formation; It is not practical to add compounds and/or change the culture medium; Risk of droplet dehydration; Intense work/ time for spheroid formation; Difficulty in scale-up.	[70,71,72,73,74,114,132,133]
	3. Magnetic levitation and Magnetic 3D printing 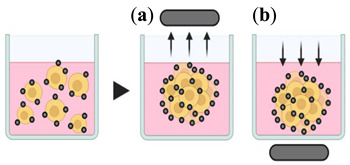	Easy development of heterotypic spheroids; Fast spheroid formation.	Require the preparation of magnetic particles; Limited spheroid formation.	[70,72,78,134]
**Scaffold-based techniques**	4. Spinner (top) and rotating (bottom) flasks 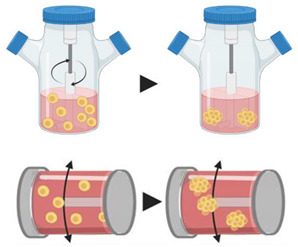	Large-scale spheroid production.	Slow agitation speed generates cell dispersion; High agitation speed generates shear force, damaging the cells; Constant agitation prevents cell visualization; Formation of spheroids with heterogeneous diameters; Requires specialized equipment.	[70,71,72,135,136]
	5. Micropatterned plates 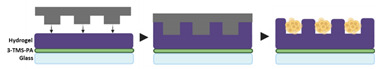	Large-scale spheroid production; Few well-to-well and plate-to-plate variation.	Formation of spheroids with heterogeneous diameters; Bubble formation during the culture; Pipetting can damage micropattern surfaces.	[64,70,93,137,138]
	6. Matrix encapsulation 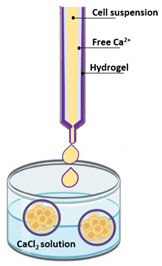	Enables cell–cell and cell–ECM interaction.	High risk of necrosis due to cell confinement.	[70,94,95,122]
	7. Matrix-on top and Matrix embedded 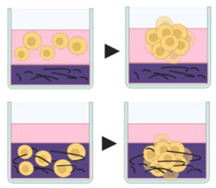	No specialized equipment required; Ease of obtaining spheroid images.	Requires prior preparation and specialized matrix handling.	[70,96,124,139]
	8. Microcarrier beads 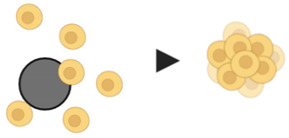	Fast, easy and reproducible method; Can form heterotypic spheroids.	Does not resemble the physiological tumor conditions in vivo.	[97,98,99,100]
	9. Microfluidic devices 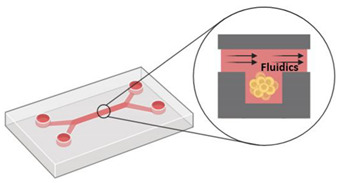	Mimic tumor vasculature.	Specialized equipment required; PDMS devices can change the flow speed when under high pressure; PDMS microfluidic can swell when exposed to some fluids; Require trained users.	[101,102,104,105,106]

## 5. Tools to Evaluate Targeting Effect

Several techniques are available to characterize spheroids, either in viable, fixed, or dissociated state, before and after anticancer drug treatment. These techniques were described in details in a number of excellent review papers [63,140,141], and allow the characterization of the organization, size, shape, gene and protein expression, metabolic status, migration and invasion of anticancer drug-treated spheroids. In general, standard biological assays used for 2D culture can be applied to spheroids, with some drawbacks as outlined below (Table 4).

### 5.1. Optical Microscopy

Morphologic changes such as size and shape can be monitored over time by optical microscopy and analyzed by appropriate software [142,143]. For instance, with a standard phase-contrast microscope, the difference in the size or volume between treated and untreated spheroids at a defined endpoint, or even during treatment, can be used to evaluate the efficacy of an anticancer drug.

Fluorescence microscopy can provide information on ECM deposition in spheroids immunostained with antibodies against fibronectin, laminin, and collagen IV [144], while relevant information such as cytoskeletal arrangement, proliferation, and apoptosis in the spheroids can be obtained by Hoechst or DAPI, phalloidin, Ki-67, caspases, Annexin V, Propidium iodide, and TUNEL staining [63,145]. Confocal laser microscopy is required to obtain higher spatial resolution, needed to analyze spheroid architecture. However, this analysis is restricted to small spheroids due to limited light penetration and to light scattering in thick tissues [143].

To overcome these issues, spheroids can be processed for histological sectioning. Then, staining methods such as hematoxylin and eosin staining allow distinction of pyknotic nuclei and eosinophilic cytoplasm in spheroid sections. For proliferating and quiescent cell populations, the use of specific antibodies in immunohistological staining is required. However, spheroid fixation used in the histological procedure precludes the study of dynamic alterations in the spheroids over time. Additionally, sample fracture and morphology deformation can occur during spheroid sectioning. Due to the delicate nature and small size of spheroids, the fixation time may need to be reduced, comparatively to biopsies or organ fragments. Further spheroid processing has also presented some challenges. For example, the inclusion of several spheroids in a unique paraffin block may involve a more arduous and costly sectioning process, since the spheroids will localize in different section planes. The development of microwell-containing apparatuses facilitated this process, allowing the simultaneous analysis of multiple spheroids in a more organized and cheaper manner [146].

To overcome these drawbacks of spheroid fixation and sectioning, faster and noninvasive microscopy approaches have been developed in the last years to image the innermost layer of live and fixed spheroids, such as light sheet fluorescence microscopy (LSFM), single or selective plane illumination microscopy (SPIM), and multi-photon microscopy (MPM) [147,148,149]. These new microscopic approaches allow deep tissue imaging study without the need of physical sectioning, while allowing dynamic processes to be studied in live 3D cultures at high resolution, under reduced light exposure and phototoxicity.

### 5.2. Electron Microscopy

Electron microscopy techniques are widely used to characterize spheroids because they provide high resolution, at nanoscale levels. High-resolution images of the internal structures can be generated by transmission electron microscopy (TEM) while high-resolution images of the surface of spheroids can be achieved by scanning electron microscopy (SEM) [56].

The TEM technique provides information on cell–cell interaction in the spheroids, such as cell junctions, and ECM deposition, as well as information on treatment outcomes such as apoptosis, cell shrinkage and organelle swelling [150]. Importantly, TEM is mostly used to analyze the distribution of drugs or nanoparticles in the spheroid [151].

The SEM technique provides high-resolution images and is used to analyze, for instance, cellular protrusions, integrity of cell–cell interactions, integrity of cellular membrane after anticancer drug treatment [56,152,153].

Both TEM and SEM are very informative although specimen collapse and morphological alterations can be associated with the steps involved in the procedures [154].

### 5.3. Flow Cytometry

Quantitative measurements such as cell viability, proliferation kinetics, cell cycle, apoptosis, and uptake of anticancer drugs and nanomedicines in spheroids can be performed using flow cytometry. Mechanical or enzymatic disaggregation of spheroids by trypsin or less toxic enzyme cocktail (Accutase^®^) is needed to obtain single cell suspension that can be stained and manipulated similarly to 2D cultures, and analyzed by flow cytometry [155]. For instance, single cells can be stained with calcein and ethidium to evaluate live cells and dead cells, respectively [56]. Other fluorescent dyes are used to analyze proliferating or quiescent cells (e.g., Propidium iodide), entry intro S phase of the cell cycle (e.g., 5-bromo-2′-deoxyuridine (BrdU) detected by a fluorescently labeled secondary antibody), or the expression of specific cellular proteins with fluorescently labelled antibodies. Flow cytometry analysis does not enable evaluation of penetration of anticancer drugs due to spheroid disaggregation and cell dissociation. However, it was reported that Hoechst 33342 (a fluorescent DNA dye) forms a marked diffusion gradient into the inner space of spheroids, therefore enabling cells of the different layers to be sorted on the basis of Hoechst staining intensity [156,157]. One major limitation of flow cytometry analysis is the need of a large amount of spheroids due to loss of cells during the process of cell dissociation [140].

### 5.4. Colorimetric Methods

Cell viability in the spheroids can be evaluated without the need of cell dissociation. For this purpose, are used colorimetric, fluorometric and luminescent methods that include acid phosphatase assay, Alamar blue, MTT assay, and lactate dehydrogenase quantification [140,158,159]. Nowadays, specialized kits for cytotoxicity assessment in spheroids are made available from many manufacturers. For instance, commercially available cell viability assays such as CellTiter-Glo 3D with better penetration of the reagents into the spheroids are easy to implement, and enable more accurate cytotoxicity determination [142,160]

### 5.5. Molecular Biology Tools

Standard molecular biology assays such as Western blot and qRT-PCR are useful to evaluate differential protein and gene expression, respectively, between 2D and 3D systems and/or before and after drug treatment. These techniques involve the use of cell lysis during the procedures of cellular protein and RNA extraction from the spheroids [59,161].

**Table 4:** Methods currently used to characterize spheroids and to evaluate drug effect.

Method		Description	Staining Methods/Markers	Feature Evaluated	Advantages (↑) and Limitations (↓)	References
Phase contrast microscopy		Monitorization of morphology and general state of spheroids.	-	Size/volume and shape.	↑ Low cost and easy method to observe the general data on spheroids size and shape. ↑ Noninvasive. ↓ Does not provide enough quality in focus to obtain detailed data from complex 3D spheroid structures.	[140,142,162]
Fluorescence microscopy		Uses fluorescent dyes to analyze specific structures in the sample; Monitorization of stained/immunostained spheroids or spheroid sections.	DNA staining by Hoechst or DAPI.	DNA, nucleus.	↑ Allows easy monitoring of a wide range of features. ↓ For larger spheroids, processing for histological sectioning is required—spheroid fixation used in the histological procedure precludes the study of dynamic alterations in the spheroids over time.	[144,145,158,163,164,165,166,167]
			Fibronectin, laminin, and collagen IV staining.	ECM deposition.		
			Phalloidin staining.	cytoskeletal arrangement.		
			Ki-67 staining.	Cell proliferation.		
			Caspase staining. Annexin V + propidium iodide (PI), and TUNEL staining methods.	Cell death, apoptosis.		
			Calcein + ethidium homodimer-1 (EthD-1).	Live/cell death assays.		
Bright field microscopy		Light is transmitted through the sample, and denser areas attenuate light transmission, originating contrast.	e.g., hematoxylin and eosin staining.	Distinction of nuclei and cytoplasmic structures.	↑ Low-cost method that offers a general overview of the sample structure (of a section). ↓ Requires spheroid processing for histological sectioning.	[143,168,169,170]
Confocal laser microscopy		The use of a focused laser spot with the removal of the out-of-focus light allows to acquire higher spatial resolution images.	Same markers described for fluorescence microscopy.	Spheroid architecture.	↑ High resolution data. ↑ 3D reconstruction. ↓ Restricted to small spheroids due to limited light penetration and to light scattering in thick tissues.	[143,171,172]
				The features described for fluorescence microscopy can also be evaluated.		[173,174,175]
Light sheet fluorescence microscopy (LSFM) and single or selective plane illumination microscopy (SPIM)		High resolution data from thick experiments through the use of planar illumination incident orthogonally to the direction of observation.	Same markers described for fluorescence microscopy.	The innermost layer of live and fixed spheroids.	↑ High spatial resolution. ↑ 3D reconstruction. ↑ Noninvasive. ↑ Does not require physical sectioning. ↑ Reduced light exposure and phototoxicity. ↓ LSFM may imply high processing time and memory in order to produce high-resolution 3D images; scattering and absorption of light may limit the penetration into specimens, although some efforts have been recently made to improve those issues. ↓ The upgrading of conventional microscopes to LSFM and/or SPIM technology may be complex and, in some cases, the optical sectioning capability may be limited. ↓ Some MPM limitations have been reported, such as weak endogenous signal strength, limited imaging materials, insufficient imaging depth.	[147,148,149,175,176,177,178,179,180] _ [181,182,183,184]
Multi-photon microscopy (MPM)		MPM pulsed long wavelength is used to excite fluorophores—two photon absorption-based fluorescence.				
Electron microscopy	Scanning electron microscopy (SEM)	The surface of the structures in the sample are scanned with a beam of electrons. The emitted signals provide high-resolution images of the surface of spheroids.	-	Cellular protrusions; Integrity of cell–cell interactions; Integrity of cellular membrane after anticancer drug treatment.	↑ High resolution. ↓ In some cases, specimen collapse and morphological alterations can be associated with the steps involved in the procedures.	[56,152,153,154,185,186,187,188]
	Transmission electron microscopy (TEM)	A beam of electrons hits the sample; part of the beam is transmitted through the specimen and used to generate high resolution images; information on cell–cell interactions is provided	-	Cell junctions and ECM deposition; Drug treatment outcomes such as apoptosis, cell shrinkage and organelle swelling; Distribution of drugs or nanoparticles in the spheroid.		
Flow cytometry		Analysis of physical and chemical properties of single cells. Mechanical or enzymatic disaggregation of spheroids is required	AnnexinV/PI	Cell death, apoptosis.	↑ Quantitative analysis. ↑ After disaggregation, samples can be manipulated similarly to 2D cultures. ↓ A large amount of spheroids are required due to loss of cells during the process of cell dissociation.	[189,190,191]
			PI/ribonuclease	Cell cycle analysis.		[56,192,193]
			5-bromo-2′-deoxyuridine (BrdU) + PI (or analog).	Cell cycle analysis, quiescent cells.		[194,195]
			Calcein + ethidium homodimer-1 (EthD-1) (PI analog).	Live/dead cell analysis, detection of quiescent cells.		[56]
			Hoechst 33342	DNA staining intensity dependent on the depth of cells in the spheroid.		[156,157,196]
			Fluorescent staining of specific cellular proteins.			[197,198]
Quantitative methods for cell viability analysis	MTT	Colorimetric Evaluation of the metabolic activity through tetrazolium salt reduction.			↑ Well-known methods so far implemented for 2D culture approaches. ↓ Limited efficacy in 3D spheroids and microtissues, due to difficulties of reagents to cross cell–cell junctions and/or 3D matrices.	[140,158,159,199,200,201]
	Lactate dehydrogenase quantification	Colorimetric Cytotoxicity evaluation through the quantification of lactate dehydrogenase (LDH) release.				
	Alamar blue	Fluorometric Evaluation of the metabolic activity through ATP measurement by resazurin reduction.				
	Acid phosphatase assay (ACP)	Colorimetric Cytotoxicity evaluation through measurement of ACP activity.			↑ Highly sensitive. ↑ Does not require spheroid dissociation. ↓ Complete removal of culture medium is required, which may not be practical and increases spheroid damage risk.	[201,202,203]
	CellTiter-Glo 3D	Luminescent Evaluation of the metabolic activity through ATP measurement, by luciferin oxidation.	.		↑ Better penetration of the reagents into the spheroids. ↑ Enables higher accuracy and reproducibility in large spheroids. ↑ Does not require removal of culture medium. ↓ ATP output may be affected by several factors and is not always proportional to cell number.	[142,204,205,206,207]
Molecular biology methods for quantification of gene expression	qRT-PCR	Quantification of gene expression at mRNA level.	-		↑ Accurate and well-known methods so far implemented for 2D culture models. ↑ After disaggregation, samples can be manipulated similarly to 2D cultures. ↓ Mechanical disruption and association with chemical buffers are required to extract proteins and RNA from the cells.	[59,161,208,209,210,211]
	Western blot	Quantification of gene expression at protein level.	-			

## 6. Application of 3D Cultures in Anti-Cancer Drug Discovery and Delivery

The capacity to reproduce the in vivo 3D tumor environment such as cellular heterogeneity, gene expression patterns, cell differentiation, generation of hypoxia, activation of cell signaling pathways, and cell–cell and cell–ECM adhesions, are amongst the many advantages that prompted the use of spheroids for in vitro evaluation of chemoresistance, migration and invasion, and other aspects of tumor biology (e.g., cancer stem cells/tumorigenicity, hypoxia and tumor metabolism). We will focus on chemoresistance and migration/invasion, and provide a brief overview on the use of spheroids to study drug delivery. Details of the other aspects were reviewed elsewhere [64,70,212,213].

### 6.1. Chemoresistance

Drug resistance is a major concern responsible for the failure of the current chemotherapeutics and their ability to fight cancer, especially in aggressive and highly metastatic tumors. It is now well established that cancer cells, grown in vitro as 3D spheroids, more accurately mimic the drug behavior in terms of sensibility and resistance than cells grown as 2D monolayers [214]. This difference is probably due to the TME and the spatial organization of the spheroids [215]. Increased cell–cell and cell–matrix adhesions may lead to changes in gene expression. Upregulation of cell–adhesion molecules, such as lumican, SNED1, DARP32, and miR-146a, was reported to increase chemotherapeutic resistance in pancreatic tumor spheroids as compared to 2D monolayers [59]. Fibronectin protected DU145 prostate cancer cell spheroids against ceramide and docetaxel-induced apoptosis through interaction with Insulin like growth factor-1 receptor [216]. A variety of apoptotic stimuli, including combinations of tumor necrosis factor-related apoptosis-inducing ligand (TRAIL), ribotoxic stressors, histone deacetylase, and proteasome inhibitors, were reported to be highly effective against mesothelioma cells when grown as monolayers than when grown as multicellular spheroids [214].

Increased resistance to chemotherapeutic drugs in spheroids is attributed to many factors associated with their constitution and organization, such as hypoxia, altered cellular energy metabolism, the acidic microenvironment, the cellular heterogeneity including the presence of cancer stem cells, and cell–cell and cell–ECM interactions [215,217,218,219,220,221,222]. The mechanisms by which these factors confer chemoresistance to spheroids were nicely reviewed in [223]. While most studies showed that cells in spheroids are more chemoresistant than cells in 2D monolayers, some studies reported that cells in MCTS are equally or even more sensitive to anticancer agents than their 2D monolayer counterparts. For example, the proteasome inhibitor PS-341 was shown to be equally effective in killing ovarian and prostate tumor cells grown in the form of multicellular spheroids, and tumor cells grown in monolayer cell culture [224].

A number of studies reported that spheroids are also more radioresistant than 2D monolayers. For instance, increased cell compaction increased the resistance of human colon adenocarcinoma spheroids to ionizing radiation [225]. Besides the aforementioned factors, radioresistance may be due to decreased radiation-induced DNA damage as a consequence of lack of oxygen in the spheroids, given that oxygen seems to be required to stabilize DNA damage upon radiation [226,227,228].

### 6.2. Migration and Invasion

The acquisition of motility and migratory ability is an important hallmark of malignant tumors. Common characteristics of solid tumors, such as hypoxia and soluble mediators-mediated interactions with stromal cells, drive tumor cell migration and invasion, through essential steps that involve, amongst others, actin cytoskeleton remodeling, changes in cell–cell and cell–ECM adhesion, and protein degradation of the surrounding ECM [229,230]. Therefore, the success of studying the multistep process of metastasis relies on a 3D microenvironment through which tumor cells can move and disseminate. In this sense, tumor spheroids are viewed as relevant in vitro models for studying invasion and migration processes [70,166,231,232]. For instance, 3D spheroids display adhesion and ECM molecule expression pattern similar to that of the tumor in vivo, and can also induce expression of proteins associated with metastasis [70,231,233]. Importantly, non-tumor cells are also present in the TME and continuously interact, through paracrine signaling, with cancer cells. For instance, fibroblasts were shown to promote contact-dependent cancer cell motility and invasion of 3D spheroids in co-culture with colorectal cancer cells, a finding validated in vivo [234]. Therefore, ideal migration/invasion assays should be performed in 3D co-cultures that also include non-tumor cells, such as macrophages, dendritic cells, endothelial cells, CAFs and immune cells, in order to better simulate the migration and invasion process found in tumor tissues. CAFs, through the release of cytokines and growth factors, together with the other stromal cells, promote the epithelial-mesenchymal transition in heterotypic 3D cell cultures, resulting in tumor development and metastasis [111,234,235,236]. At the same time, endothelial cells in 3D co-cultures tend to accumulate in the peripheral layer, facilitating the adhesion and infiltration of immune cells [28]. In fact, immune cells can secrete interleukin 6 and MMP-9, which cause inflammation, angiogenesis and ECM degradation, thereby promoting tumor invasion and metastasis [237].

Several assays are available to determine the invasion and migration potential of cells in spheroids [70,232]. In the transwell-based or Boyden chamber assays, the spheroids are seeded on the top of a filter coated with a thick layer of ECM-derived components, usually collagen I, and invasion, in response to a chemo-attractant such as growth factors, can be measured by determining the number of cells that move from the top chamber to the lower chamber [70,232,238]. Additionally, the ability of the cells to invade cellular barriers can be determined by adding a layer of fibroblasts or endothelial cells on top of the matrix [70]. This latter is particularly relevant to mimic the ability of cancer cells to cross the blood vessel barrier and to invade deeply the tissues. Alternatively, spheroids can be completely embedded into different matrices, usually between two layers of ECM gel, where cells leave the spheroids and invade the surrounding matrix [96,239]. Sophisticated techniques combined with computerized quantification are now available to reproducibly perform optimized experimental conditions and to calculate the invasive index of cells [70,239,240,241]. For instance, the extent and rate of tumor spheroid invasion, using the 3D spheroid invasion assay, was rapidly and reproducibly measured using imaging cytometer [238]. Spheroid invasion assays can also be used as a metric to measure drug efficacy [96]. For example, lower concentrations of the adjuvant gamma-linolenic acid caused an increase in glioma spheroid invasion, but increased the apoptotic index at higher concentrations [242]. In sum, spheroids have been widely utilized to study the role of mechanisms involved in cellular invasion, and represent a valuable tool for preclinical evaluation of therapeutic agents targeting invasion [96,166,232].

### 6.3. Spheroids and Nanomedicines

Systemic drug toxicity and poor efficacy remain a major concern in cancer therapy due to the lack of selective drug delivery to tumor tissues, stressing the need to improve tumor targeting [243]. Nanomedicines have thus emerged as promising approach to (actively) target tumor and improve drug delivery. These nanostructures are biocompatible, biodegradable, non-toxic, can be prepared on a large scale, can provide controlled drug release, and enhance tissue/cell-specific targeting, in addition to reducing side effects [244,245,246,247,248]. However, despite the promising preclinical outcome that was reported for a significant number of nanotherapeutics, only few nanodrugs reached the clinic and achieved the expected results in patients [243]. Many barriers influence the efficiency of nanomedicine delivery to the target tumor, that are not recapitulated by the 2D monolayer cultures.

Tissue penetration of nanoparticles (NPs) relies on their diffusion capacity through the ECM, which varies in density and size, and is also influenced by cell–cell interactions, necrotic core, hypoxia, and by the intravascular pressure irregularities due to vessel compressions applied by growing tumors [249,250,251]. In this sense, as outlined above, spheroids have gained in popularity over traditional 2D culture systems because their pathophysiological features are close to those of the native tumors, being an excellent model to evaluate nanodrugs and to better predict their clinical outcomes [101,197,212,252]. Consequently, spheroids have been used as valuable tool to study different physico-chemical proprieties of nanocarriers such as chemical composition, size, shape and surface properties, which are crucial for their penetration and antitumor efficacy [197,253,254].

A general observation from studies that used spheroids is that nanoparticles (NPs) penetration is inversely correlated to the particle size [159,254,255,256]. NPs with small size (<100 nm) penetrate deeply and faster in the spheroids and distribute homogeneously, as compared to larger NPs (>100 nm) which remain confined to the superficial layers [159,257,258,259]. However, NPs <50 nm were reported to interact with liver cells, and to be poorly retained in the tumor [260].

The surface charge of NPs also influences their penetration in the spheroids: negatively charged NPs penetrate deeply while their positive counterparts remain at the outer layers [56,199]. Yet, more effective drug delivery is warranted by NPs with positive surface charge due to electrostatic interactions with negatively charged cell membranes. To overcome this issue, it has been proposed the use of pH-responsive negatively charged NPs that can turn to positively charged ones once in contact with acidic conditions (e.g., tumor microenvironment), so that negative surface charge ensures deep penetration in the spheroids, while positive surface charge enables more effective drug delivery [199,261].

Although little information exists on the influence of NP shape on penetration and accumulation in the spheroid, the existing literature indicates that nanorods seem to diffuse more rapidly in spheroids compared to nanospheres, and that short nanorods (400 nm in length) accumulate more rapidly and are better internalized than long nanorods (<2000 nm in length) [262,263,264].

Interestingly, NP penetration into spheroids has been enhanced by modification of the surface coating. For instance, ECM-degrading enzymes such as collagenases have been used to coat NPs of up to 100 nm in size, which demonstrated superior (4-fold increase) penetration over control NPs [258]. Drug efficacy is the most important endpoint of any formulation, and it depends greatly on the penetration and accumulation into the spheroids [254]. In general, nanocarrier formulations with high penetration and accumulation in the spheroids exhibited better antitumor activity [159].

Comparison between NP delivery and efficacy between 3D tumor spheroids and animal models revealed key similarities between the two systems. For instance, the photosensitizer verteporfin encapsulated into lipid nanocarriers strongly reduced tumor cell viability of ovarian spheroid cancer cells, and also inhibited tumor growth in an orthotopic murine ovarian cancer model, when compared to free drug [265]. Similar to in vivo tissues, HepG2 cells in 3D hydrogels were more resistant to biotin-conjugated pullulan acetate nanoparticles (Bio-PA NPs) treatments compared to the 2D system [266]. Moreover, Bio-PA NPs exhibited similar anti-tumor activity in 3D culture cells and in in vivo xenografted hepatic tumor model [266]. Studies also observed that iRGD-conjugated nanoparticles with doxorubicin were able to accumulate with more efficacy and penetrate deeply into tumor in both SH-SY5Y spheroids and H22 tumor-bearing mice, restraining tumor growth in both systems [267]. Overall, this highlights the predictive power of spheroids for in vivo therapeutic efficacy, and their potential as promising alternative to animal models for cancer study, hopefully resolving high cost and ethical issues associated with animal use.

## 7. Concluding Remarks and Perspectives

It is consensual that 3D tumor models enable evaluation of anticancer drugs and nanomedicines in a condition closer to the real tumor, owing to their key features such as spatial organization, cell–cell and cell–ECM, diffusive gradients, complex cell signaling, drug resistance and metabolic adaptation. As reviewed here, these features are missing in 2D culture systems and, consequently, 3D culture models in preclinical evaluation are expected to provide more accurate results of the therapeutic potential of anticancer drug candidates, thereby increasing the predictability of the in vivo efficacy. Identifying and eliminating those therapeutics that did not show any interesting efficacy in 3D cultures will reduce animal use and speed up the number of therapeutics that reach the clinic.

It is noteworthy that most of the published works used spheroids made of only cancer cells, and, thus, do not represent the complexity associated with the diversity of the cellular and non-cellular components present in the real tumor. Spheroids that incorporate cell types recapitulating the vasculature (e.g., endothelial cells), the immune system (e.g., leukocytes) and ECM production (e.g., fibroblasts) are, thus, highly recommended. This is important as it would make the geometry of drug penetration in the spheroids closely similar to that in vivo, therefore providing better prediction of drug effects and delivery mechanisms and, at the same time, reducing costly investments associated with the ultimate step of clinical investigations.

Standardized methodologies for generation and characterization of spheroids are urgently needed, this would avoid variability in size and homogeneity, as well as in biological effect evaluation. Although considerable progress has been made to adapt existing 2D culture analysis assays to the spheroid model, many challenges remain to be addressed. Enabling acquisition of high-resolution images from intact spheroids remains a major challenge, due to the size of spheroids and poor light scattering. On the other hand, histological procedures for spheroid sectioning require special care in handling, as specimen tend to collapse or fracture easily. Mass production, together with developing easy to handle spheroids that are time and cost effective, with reduced workflows of culture and analysis, is crucial in order to encourage their routine use in drug discovery research. We are, yet, still far from giving up using animal models for safety and efficacy studies of drugs. Meanwhile, and ideally, the use of spheroids in preclinical testing could reduce the number of compounds progressing to in vivo testing, thereby reducing the numbers of animals used.

In conclusion, the use of 3D models to assess tumor penetration, accumulation and antitumor activity of drug and nanomedicine candidates is becoming a reality, and should turn out a mandatory step between 2D and in vivo models in the near future, with a great impact on the transferability of new anticancer drugs from bench to bedside. Hopefully, the generation of tumor spheroids from the patient’s own cells may enable personalized approaches to screening and selecting the appropriate drugs for the patients.

## Figures and Tables

**Table 1:** Differences between conventional 2D monolayer and 3D spheroid cultures.

Cell Culture System	Advantages	Disadvantages
2D cultures	Fast replication; Low cost; Easy to manipulate; Establish long-term cultures.	Homogeneity in oxygen and nutrients perfusion; Decreased cell–cell and cell–ECM interactions; More susceptible to pharmacological action; Poor cell differentiation; Faster proliferation than in vivo tumors. Modified genetic profile when compared to in vivo tissue.
3D cultures	Heterogeneity in oxygen and nutrients perfusion; 3 different layers (proliferation, quiescence and necrosis zones) resembling the in vivo tumors; Increased cell–cell and cell–ECM interactions; Mimic drug penetration in the tumor. Recapitulate the genetic in vivo profile.	High cost; Greater difficulty in carrying out methodological techniques.

## References

[1] Hait W.N. (2010). Anticancer drug development: The grand challenges. Nat. Rev. Drug. Discov..

[2] Hutchinson L., Kirk R. (2011). High drug attrition rates—Where are we going wrong?. Nat. Rev. Clin. Oncol..

[3] Rubinstein L.V., Shoemaker R.H., Paull K.D., Simon R.M., Tosini S., Skehan P., Scudiero D.A., Monks A., Boyd M.R. (1990). Comparison of In Vitro Anticancer-Drug-Screening Data Generated With a Tetrazolium Assay Versus a Protein Assay Against a Diverse Panel of Human Tumor Cell Lines. JNCI J. Natl. Cancer Inst..

[4] Ocana A., Pandiella A., Siu L.L., Tannock I.F. (2011). Preclinical development of molecular-targeted agents for cancer. Nat. Rev. Clin. Oncol..

[5] van der Worp H.B., Howells D.W., Sena E.S., Porritt M.J., Rewell S., O’Collins V., Macleod M.R. (2010). Can animal models of disease reliably inform human studies?. PLoS Med..

[6] Quail D.F., Joyce J.A. (2013). Microenvironmental regulation of tumor progression and metastasis. Nat. Med..

[7] Belli C., Trapani D., Viale G., D’Amico P., Duso B.A., Della Vigna P., Orsi F., Curigliano G. (2018). Targeting the microenvironment in solid tumors. Cancer Treat. Rev..

[8] Imamura Y., Mukohara T., Shimono Y., Funakoshi Y., Chayahara N., Toyoda M., Kiyota N., Takao S., Kono S., Nakatsura T. (2015). Comparison of 2D- and 3D-culture models as drug-testing platforms in breast cancer. Oncol. Rep..

[9] Birgersdotter A., Sandberg R., Ernberg I. (2005). Gene expression perturbation in vitro--a growing case for three-dimensional (3D) culture systems. Semin. Cancer Biol..

[10] Souza A.G., Silva I.B.B., Campos-Fernandez E., Barcelos L.S., Souza J.B., Marangoni K., Goulart L.R., Alonso-Goulart V. (2018). Comparative Assay of 2D and 3D Cell Culture Models, Proliferation, Gene Expression and Anticancer Drug Response. Curr. Pharm. Des..

[11] Teicher B.A. (2006). Tumor models for efficacy determination. Mol. Cancer Ther..

[12] Bissell M.J. (2007). Architecture Is the Message, The role of extracellular matrix and 3-D structure in tissue-specific gene expression and breast cancer. Pezcoller Found J..

[13] Ravi M., Paramesh V., Kaviya S.R., Anuradha E., Solomon F.P. (2015). 3D cell culture systems: Advantages and applications. J. Cell Physiol..

[14] Białkowska K., Komorowski P., Bryszewska M., Miłowska K. (2020). Spheroids as a Type of Three-Dimensional Cell Cultures-Examples of Methods of Preparation and the Most Important Application. Int. J. Mol. Sci..

[15] Shehzad A., Ravinayagam V., AlRumaih H., Aljafary M., Almohazey D., Almofty S., Al-Rashid N.A., Al-Suhaimi E.A. (2019). Application of Three-dimensional (3D) Tumor Cell Culture Systems and Mechanism of Drug Resistance. Curr. Pharm. Des..

[16] Park J.I., Lee J., Kwon J.L., Park H.B., Lee S.Y., Kim J.Y., Sung J., Kim J.M., Song K.S., Kim K.H. (2016). Scaffold-Free Coculture Spheroids of Human Colonic Adenocarcinoma Cells and Normal Colonic Fibroblasts Promote Tumorigenicity in Nude Mice. Transl. Oncol..

[17] Szade K., Zukowska M., Szade A., Collet G., Kloska D., Kieda C., Jozkowicz A., Dulak J. (2016). Spheroid-plug model as a tool to study tumor development, angiogenesis, and heterogeneity in vivo. Tumour Biol. J. Int. Soc. Oncodev. Biol. Med..

[18] Zhou J., Tang Z., Gao S., Li C., Feng Y., Zhou X. (2020). Tumor-Associated Macrophages: Recent Insights and Therapies. Front. Oncol..

[19] Wei C., Yang C., Wang S., Shi D., Zhang C., Lin X., Liu Q., Dou R., Xiong B. (2019). Crosstalk between cancer cells and tumor associated macrophages is required for mesenchymal circulating tumor cell-mediated colorectal cancer metastasis. Mol. Cancer.

[20] Lin Y., Xu J., Lan H. (2019). Tumor-associated macrophages in tumor metastasis: Biological roles and clinical therapeutic applications. J. Hematol. Oncol..

[21] Garufi A., Traversi G., Cirone M., D’Orazi G. (2019). HIPK2 role in the tumor-host interaction, Impact on fibroblasts transdifferentiation CAF-like. IUBMB Life.

[22] Sahai E., Astsaturov I., Cukierman E., DeNardo D.G., Egeblad M., Evans R.M., Fearon D., Greten F.R., Hingorani S.R., Hunter T. (2020). A framework for advancing our understanding of cancer-associated fibroblasts. Nat. Rev. Cancer.

[23] Wagner J., Rapsomaniki M.A., Chevrier S., Anzeneder T., Langwieder C., Dykgers A., Rees M., Ramaswamy A., Muenst S., Soysal S.D. (2019). A Single-Cell Atlas of the Tumor and Immune Ecosystem of Human Breast Cancer. Cell.

[24] Hinshaw D.C., Shevde L.A. (2019). The Tumor Microenvironment Innately Modulates Cancer Progression. Cancer Res..

[25] Reina-Campos M., Moscat J., Diaz-Meco M. (2017). Metabolism shapes the tumor microenvironment. Curr. Opin. Cell Biol..

[26] Gonzalez H., Hagerling C., Werb Z. (2018). Roles of the immune system in cancer: From tumor initiation to metastatic progression. Genes Dev..

[27] Zhang J., Zhang Q., Lou Y., Fu Q., Chen Q., Wei T., Yang J., Tang J., Wang J., Chen Y. (2018). Hypoxia-inducible factor-1α/interleukin-1β signaling enhances hepatoma epithelial-mesenchymal transition through macrophages in a hypoxic-inflammatory microenvironment. Hepatology.

[28] Aung A., Kumar V., Theprungsirikul J., Davey S.K., Varghese S. (2020). An Engineered Tumor-on-a-Chip Device with Breast Cancer-Immune Cell Interactions for Assessing T-cell Recruitment. Cancer Res..

[29] Kashyap A.S., Schmittnaegel M., Rigamonti N., Pais-Ferreira D., Mueller P., Buchi M., Ooi C.H., Kreuzaler M., Hirschmann P., Guichard A. (2020). Optimized antiangiogenic reprogramming of the tumor microenvironment potentiates CD40 immunotherapy. Proc. Natl. Acad. Sci. USA.

[30] Protopsaltis N.J., Liang W., Nudleman E., Ferrara N. (2019). Interleukin-22 promotes tumor angiogenesis. Angiogenesis.

[31] O’Donnell J.S., Teng M.W.L., Smyth M.J. (2019). Cancer immunoediting and resistance to T cell-based immunotherapy. Nat. Rev. Clin. Oncol..

[32] Domschke C., Schneeweiss A., Stefanovic S., Wallwiener M., Heil J., Rom J., Sohn C., Beckhove P., Schuetz F. (2016). Cellular Immune Responses and Immune Escape Mechanisms in Breast Cancer, Determinants of Immunotherapy. Breast Care.

[33] Yuan Y. (2016). Spatial Heterogeneity in the Tumor Microenvironment. Cold Spring Harb. Perspect. Med..

[34] Janssen L.M.E., Ramsay E.E., Logsdon C.D., Overwijk W.W. (2017). The immune system in cancer metastasis: Friend or foe?. J. Immunother. Cancer.

[35] Choi H., Moon A. (2018). Crosstalk between cancer cells and endothelial cells: Implications for tumor progression and intervention. Arch. Pharm. Res..

[36] Houthuijzen J.M., Jonkers J. (2018). Cancer-associated fibroblasts as key regulators of the breast cancer tumor microenvironment. Cancer Metastasis Rev..

[37] Wang J.J., Lei K.F., Han F. (2018). Tumor microenvironment: Recent advances in various cancer treatments. Eur. Rev. Med. Pharmacol. Sci..

[38] Shaked Y. (2019). The pro-tumorigenic host response to cancer therapies. Nat. Rev. Cancer.

[39] Jing X., Yang F., Shao C., Wei K., Xie M., Shen H., Shu Y. (2019). Role of hypoxia in cancer therapy by regulating the tumor microenvironment. Mol. Cancer.

[40] Zhong S., Jeong J.H., Chen ZChen Z., Luo J.L. (2020). Targeting Tumor Microenvironment by Small-Molecule Inhibitors. Transl. Oncol..

[41] Luo W., Wang Y. (2019). Hypoxia Mediates Tumor Malignancy and Therapy Resistance. Adv. Exp. Med. Biol..

[42] Gandhi N., Das G.M. (2019). Metabolic Reprogramming in Breast Cancer and Its Therapeutic Implications. Cells.

[43] Wu T., Dai Y. (2017). Tumor microenvironment and therapeutic response. Cancer Lett..

[44] Thews O., Riemann A. (2019). Tumor pH and metastasis: A malignant process beyond hypoxia. Cancer Metastasis Rev..

[45] Swenson E.R. (2016). Hypoxia and Its Acid-Base Consequences, From Mountains to Malignancy. Adv. Exp. Med. Biol..

[46] White K.A., Grillo-Hill B.K., Barber D.L. (2017). Cancer cell behaviors mediated by dysregulated pH dynamics at a glance. J. Cell Sci..

[47] Paškevičiūtė M., Petrikaitė V. (2019). Proton Pump Inhibitors Modulate Transport Of Doxorubicin And Its Liposomal Form Into 2D And 3D Breast Cancer Cell Cultures. Cancer Manag. Res..

[48] Pitt J.M., Marabelle A., Eggermont A., Soria J.C., Kroemer G., Zitvogel L. (2016). Targeting the tumor microenvironment: Removing obstruction to anticancer immune responses and immunotherapy. Ann. Oncol..

[49] Jarosz-Biej M., Smolarczyk R., Cichoń T., Kułach N. (2019). Tumor Microenvironment as A “Game Changer” in Cancer Radiotherapy. Int. J. Mol. Sci..

[50] Riera-Domingo C., Audigé A., Granja S., Cheng W.C., Ho P.C., Baltazar F., Stockmann C., Mazzone M. (2020). Immunity, Hypoxia, and Metabolism-the Ménage à Trois of Cancer, Implications for Immunotherapy. Physiol. Rev..

[51] Najafi M., Farhood B., Mortezaee K. (2019). Extracellular matrix (ECM) stiffness and degradation as cancer drivers. J. Cell Biochem..

[52] Sangaletti S., Chiodoni C., Tripodo C., Colombo M.P. (2017). The good and bad of targeting cancer-associated extracellular matrix. Curr. Opin. Pharmacol..

[53] Roma-Rodrigues C., Mendes R., Baptista P.V., Fernandes A.R. (2019). Targeting Tumor Microenvironment for Cancer Therapy. Int. J. Mol. Sci..

[54] Hanahan D., Weinberg R.A. (2011). Hallmarks of cancer: The next generation. Cell.

[55] Kitaeva K.V., Rutland C.S., Rizvanov A.A., Solovyeva V.V. (2020). Cell Culture Based in vitro Test Systems for Anticancer Drug Screening. Front. Bioeng. Biotechnol..

[56] Ma H.L., Jiang Q., Han S., Wu Y., Tomshine J.C., Wang D., Gan Y., Zou G., Liang X.J. (2012). Multicellular tumor spheroids as an in vivo-like tumor model for three-dimensional imaging of chemotherapeutic and nano material cellular penetration. Mol. Imaging.

[57] Baker B.M., Chen C.S. (2012). Deconstructing the third dimension: How 3D culture microenvironments alter cellular cues. J. Cell Sci..

[58] Hirschhaeuser F., Menne H., Dittfeld C., West J., Mueller-Klieser W., Kunz-Schughart L.A. (2010). Multicellular tumor spheroids: An underestimated tool is catching up again. J. Biotechnol..

[59] Longati P., Jia X., Eimer J., Wagman A., Witt M.R., Rehnmark S., Verbeke C., Toftgård R., Löhr M., Heuchel R.L. (2013). 3D pancreatic carcinoma spheroids induce a matrix-rich, chemoresistant phenotype offering a better model for drug testing. BMC Cancer.

[60] Yamada K.M., Cukierman E. (2007). Modeling tissue morphogenesis and cancer in 3D. Cell.

[61] Goldhammer N., Kim J., Timmermans-Wielenga V., Petersen O.W. (2019). Characterization of organoid cultured human breast cancer. Breast Cancer Res..

[62] Baal N., Widmer-Teske R., McKinnon T., Preissner K.T., Zygmunt M.T. (2009). In vitro spheroid model of placental vasculogenesis: Does it work?. Lab. Investig..

[63] Costa E.C., Moreira A.F., de Melo-Diogo D., Gaspar V.M., Carvalho M.P., Correia I.J. (2016). 3D tumor spheroids: An overview on the tools and techniques used for their analysis. Biotechnol. Adv..

[64] Fang Y., Eglen R.M. (2017). Three-Dimensional Cell Cultures in Drug Discovery and Development. SLAS Discov..

[65] Gao W., Wu D., Wang Y., Wang Z., Zou C., Dai Y., Ng C.F., Teoh J.Y.C., Chan F.L. (2018). Development of a novel and economical agar-based non-adherent three-dimensional culture method for enrichment of cancer stem-like cells. Stem Cell Res. Ther..

[66] Xiang X., Phung Y., Feng M., Nagashima K., Zhang J., Broaddus V.C., Hassan R., FitzGerald D., Ho M. (2011). The development and characterization of a human mesothelioma in vitro 3D model to investigate immunotoxin therapy. PLoS ONE.

[67] Sarisozen C., Abouzeid A.H., Torchilin V.P. (2014). The effect of co-delivery of paclitaxel and curcumin by transferrin-targeted PEG-PE-based mixed micelles on resistant ovarian cancer in 3-D spheroids and in vivo tumors. Eur. J. Pharm. Biopharm..

[68] Sarisozen C., Dhokai S., Tsikudo E.G., Luther E., Rachman I.M., Torchilin V.P. (2016). Nanomedicine based curcumin and doxorubicin combination treatment of glioblastoma with scFv-targeted micelles, In vitro evaluation on 2D and 3D tumor models. Eur. J. Pharm. Biopharm..

[69] Ekert J.E., Johnson K., Strake B., Pardinas J., Jarantow S., Perkinson R., Colter D.C. (2014). Three-dimensional lung tumor microenvironment modulates therapeutic compound responsiveness in vitro--implication for drug development. PLoS ONE.

[70] Nath S., Devi G.R. (2016). Three-dimensional culture systems in cancer research, Focus on tumor spheroid model. Pharmacol. Ther..

[71] Achilli T.M., Meyer J., Morgan J.R. (2012). Advances in the formation, use and understanding of multi-cellular spheroids. Expert Opin. Biol. Ther..

[72] Verjans E.T., Doijen J., Luyten W., Landuyt B., Schoofs L. (2018). Three-dimensional cell culture models for anticancer drug screening, Worth the effort?. J. Cell Physiol..

[73] Froehlich K., Haeger J.D., Heger J., Pastuschek J., Photini S.M., Yan Y., Lupp A., Pfarrer C., Mrowka R., Schleußner E. (2016). Generation of Multicellular Breast Cancer Tumor Spheroids, Comparison of Different Protocols. J. Mammary Gland Biol. Neoplasia.

[74] Lee D., Pathak S., Jeong J.-H. (2019). Design and manufacture of 3D cell culture plate for mass production of cell-spheroids. Sci. Rep..

[75] Cho C.Y., Chiang T.H., Hsieh L.H., Yang W.Y., Hsu H.H., Yeh C.K., Huang C.C., Huang J.H. (2020). Development of a Novel Hanging Drop Platform for Engineering Controllable 3D Microenvironments. Front. Cell Dev. Biol..

[76] Wu H.-W., Hsiao Y.-H., Chen C.-C., Yet S.F., Hsu C.H. (2016). A PDMS-Based Microfluidic Hanging Drop Chip for Embryoid Body Formation. Molecules.

[77] Zhao L., Xiu J., Liu Y., Zhang T., Pan W., Zheng X., Zhang X. (2019). A 3D Printed Hanging Drop Dripper for Tumor Spheroids Analysis Without Recovery. Sci. Rep..

[78] Ryu N.E., Lee S.H., Park H. (2019). Spheroid Culture System Methods and Applications for Mesenchymal Stem Cells. Cells.

[79] Rijal G., Li W. (2017). A versatile 3D tissue matrix scaffold system for tumor modeling and drug screening. Sci. Adv..

[80] Kuriakose A.E., Hu W., Nguyen K.T., Menon J.U. (2019). Scaffold-based lung tumor culture on porous PLGA microparticle substrates. PLoS ONE.

[81] Zhang M., Boughton P., Rose B., Lee C.S., Hong A.M. (2013). The use of porous scaffold as a tumor model. Int. J. Biomater..

[82] Xiao Y., Zhou M., Zhang M., Liu W., Zhou Y., Lang M. (2019). Hepatocyte culture on 3D porous scaffolds of PCL/PMCL. Colloids Surf. B Biointerfaces.

[83] Wang X., Dai X., Zhang X., Li X., Xu T., Lan Q. (2018). Enrichment of glioma stem cell-like cells on 3D porous scaffolds composed of different extracellular matrix. Biochem. Biophys. Res. Commun..

[84] Vasanthan K.S., Subramaniam A., Krishnan U.M., Sethuraman S. (2015). Influence of 3D porous galactose containing PVA/gelatin hydrogel scaffolds on three-dimensional spheroidal morphology of hepatocytes. J. Mater. Sci. Mater. Med..

[85] Florczyk S.J., Wang K., Jana S., Wood D.L., Sytsma S.K., Sham J.G., Kievit F.M., Zhang M. (2013). Porous chitosan-hyaluronic acid scaffolds as a mimic of glioblastoma microenvironment ECM. Biomaterials.

[86] De T., Goyal S., Balachander G., Chatterjee K., Kumar P., Babu K.G., Rangarajan A. (2019). A Novel Ex Vivo System Using 3D Polymer Scaffold to Culture Circulating Tumor Cells from Breast Cancer Patients Exhibits Dynamic E-M Phenotypes. J. Clin. Med..

[87] Wang K., Kievit F.M., Florczyk S.J., Stephen Z.R., Zhang M. (2015). 3D Porous Chitosan-Alginate Scaffolds as an In Vitro Model for Evaluating Nanoparticle-Mediated Tumor Targeting and Gene Delivery to Prostate Cancer. Biomacromolecules.

[88] Bäcker A., Erhardt O., Wietbrock L., Schel N., Göppert B., Dirschka M., Abaffy P., Sollich T., Cecilia A., Gruhl F.J. (2017). Silk scaffolds connected with different naturally occurring biomaterials for prostate cancer cell cultivation in 3D. Biopolymers.

[89] Fischbach C., Chen R., Matsumoto T., Schmelzle T., Brugge J.S., Polverini P.J., Mooney D.J. (2007). Engineering tumors with 3D scaffolds. Nat. Methods.

[90] Zhang J., Wehrle E., Vetsch J.R., Paul G.R., Rubert M., Müller R. (2019). Alginate dependent changes of physical properties in 3D bioprinted cell-laden porous scaffolds affect cell viability and cell morphology. Biomed. Mater..

[91] Alghuwainem A., Alshareeda A.T., Alsowayan B. (2019). Scaffold-Free 3-D Cell Sheet Technique Bridges the Gap between 2-D Cell Culture and Animal Models. Int. J. Mol. Sci..

[92] Gong X., Lin C., Cheng J., Su J., Zhao H., Liu T., Wen X., Zhao P. (2015). Generation of Multicellular Tumor Spheroids with Microwell-Based Agarose Scaffolds for Drug Testing. PLoS ONE.

[93] Sant S., Johnston P.A. (2017). The production of 3D tumor spheroids for cancer drug discovery. Drug Discov. Today Technol..

[94] Ferreira L.P., Gaspar V.M., Mano J.F. (2018). Design of spherically structured 3D in vitro tumor models -Advances and prospects. Acta Biomater..

[95] Huang X., Zhang X., Wang X., Wang C., Tang B. (2012). Microenvironment of alginate-based microcapsules for cell culture and tissue engineering. J. Biosci. Bioeng..

[96] Tevis K.M., Colson Y.L., Grinstaff M.W. (2017). Embedded Spheroids as Models of the Cancer Microenvironment. Adv. Biosyst..

[97] Akins R.E., Schroedl N.A., Gonda S.R., Hartzell C.R. (1997). Neonatal rat heart cells cultured in simulated microgravity. In Vitro Cell Dev. Biol. Anim..

[98] Antoni D., Burckel H., Josset E., Noel G. (2015). Three-dimensional cell culture: A breakthrough in vivo. Int. J. Mol. Sci..

[99] Huang L., Abdalla A.M.E., Xiao L., Yang G. (2020). Biopolymer-Based Microcarriers for Three-Dimensional Cell Culture and Engineered Tissue Formation. Int. J. Mol. Sci..

[100] Liu H., Lu T., Kremers G.-J., Seynhaeve A.L., ten Hagen T.L. (2020). A microcarrier-based spheroid 3D invasion assay to monitor dynamic cell movement in extracellular matrix. Biol. Proced. Online.

[101] Mehta G., Hsiao A.Y., Ingram M., Luker G.D., Takayama S. (2012). Opportunities and challenges for use of tumor spheroids as models to test drug delivery and efficacy. J. Control. Release.

[102] Vadivelu R.K., Kamble H., Shiddiky M.J.A., Nguyen N.-T. (2017). Microfluidic Technology for the Generation of Cell Spheroids and Their Applications. Micromachines.

[103] Chen C., Townsend A.D., Hayter E., Birk H.M., Sell S.A., Martin R.S. (2018). Insert-based microfluidics for 3D cell culture with analysis. Anal. Bioanal. Chem..

[104] Kuriu S., Kadonosono T., Kizaka-Kondoh S., Ishida T. (2020). Slicing Spheroids in Microfluidic Devices for Morphological and Immunohistochemical Analysis. Micromachines.

[105] Tanyeri M., Tay S. (2018). Viable cell culture in PDMS-based microfluidic devices. Methods Cell Biol..

[106] Hunter L., Gala de Pablo J., Stammers A.C., Thomson N.H., Evans S.D., Shim J.U. (2020). On-chip pressure measurements and channel deformation after oil absorption. SN Appl. Sci..

[107] Kuncová-Kallio J., Kallio P.J. (2006). PDMS and its suitability for analytical microfluidic devices. Conf. Proc. IEEE Eng. Med. Biol. Soc..

[108] Martin A., Teychené S., Camy S., Aubin J. (2016). Fast and inexpensive method for the fabrication of transparent pressure-resistant microfluidic chips. Microfluid. Nanofluidics.

[109] Kochanek S.J., Close D.A., Johnston P.A. (2019). High Content Screening Characterization of Head and Neck Squamous Cell Carcinoma Multicellular Tumor Spheroid Cultures Generated in 384-Well Ultra-Low Attachment Plates to Screen for Better Cancer Drug Leads. Assay Drug Dev. Technol..

[110] Close D.A., Camarco D.P., Shan F., Kochanek S.J., Johnston P.A. (2018). The Generation of Three-Dimensional Head and Neck Cancer Models for Drug Discovery in 384-Well Ultra-Low Attachment Microplates. Methods Mol. Biol..

[111] Khawar I.A., Park J.K., Jung E.S., Lee M.A., Chang S., Kuh H.J. (2018). Three Dimensional Mixed-Cell Spheroids Mimic Stroma-Mediated Chemoresistance and Invasive Migration in hepatocellular carcinoma. Neoplasia.

[112] Kim M., Yun H.-W., Choi B.H., Min B.H. (2018). Three-Dimensional Spheroid Culture Increases Exosome Secretion from Mesenchymal Stem Cells. Tissue Eng. Regen. Med..

[113] Hurrell T., Ellero A.A., Masso Z.F., Cromarty A.D. (2018). Characterization and reproducibility of HepG2 hanging drop spheroids toxicology in vitro. Toxicol. In Vitro.

[114] Djomehri S.I., Burman B., Gonzalez M.E., Takayama S., Kleer C.G. (2019). A reproducible scaffold-free 3D organoid model to study neoplastic progression in breast cancer. J. Cell Commun. Signal..

[115] Perez J.E., Nagle I., Wilhelm C. (2020). Magnetic molding of tumor spheroids: Emerging model for cancer screening. Biofabrication.

[116] Mishriki S., Abdel Fattah A.R., Kammann T., Sahu R.P., Geng F., Puri I.K. (2019). Rapid Magnetic 3D Printing of Cellular Structures with MCF-7 Cell Inks. Research.

[117] Urbanczyk M., Zbinden A., Layland S.L., Duffy G., Schenke-Layland K. (2020). Controlled Heterotypic Pseudo-Islet Assembly of Human β-Cells and Human Umbilical Vein Endothelial Cells Using Magnetic Levitation. Tissue Eng. Part A.

[118] Casson J., O’Kane S., Smith C.-A., Dalby M.J., Berry C.C. (2018). Interleukin 6 Plays a Role in the Migration of Magnetically Levitated Mesenchymal Stem Cells Spheroids. Appl. Sci..

[119] Pawlik T.M., Souba W.W., Sweeney T.J., Bode B.P. (2000). Amino acid uptake and regulation in multicellular hepatoma spheroids. J. Surg. Res..

[120] Yakavets I., Yankovsky I., Millard M., Lamy L., Lassalle H.P., Wiehe A., Zorin V., Bezdetnaya L. (2017). The alteration of temoporfin distribution in multicellular tumor spheroids by β-cyclodextrins. Int. J. Pharm..

[121] Itaka K., Uchida S., Matsui A., Yanagihara K., Ikegami M., Endo T., Ishii T., Kataoka K. (2015). Gene Transfection toward Spheroid Cells on Micropatterned Culture Plates for Genetically-modified Cell Transplantation. J. Vis. Exp. JoVE.

[122] Alessandri K., Sarangi B.R., Gurchenkov V.V., Sinha B., Kießling T.R., Fetler L., Rico F., Scheuring S., Lamaze C., Simon A. (2013). Cellular capsules as a tool for multicellular spheroid production and for investigating the mechanics of tumor progression in vitro. Proc Natl Acad Sci USA.

[123] Heo D.N., Hospodiuk M., Ozbolat I.T. (2019). Synergistic interplay between human MSCs and HUVECs in 3D spheroids laden in collagen/fibrin hydrogels for bone tissue engineering. Acta Biomater..

[124] Utama R.H., Atapattu L., O’Mahony A.P., Fife C.M., Baek J., Allard T., O’Mahony K.J., Ribeiro J.C., Gaus K., Kavallaris M. (2020). A 3D Bioprinter Specifically Designed for the High-Throughput Production of Matrix-Embedded Multicellular Spheroids. iScience.

[125] Tevis K.M., Cecchi R.J., Colson Y.L., Grinstaff M.W. (2017). Mimicking the tumor microenvironment to regulate macrophage phenotype and assessing chemotherapeutic efficacy in embedded cancer cell/macrophage spheroid models. Acta Biomater..

[126] Jianmin Z., Hongfang W., Meifu F. (2002). Resistance of multicellular aggregates to pharmorubicin observed in human hepatocarcinoma cells. Braz. J. Med. Biol. Res..

[127] Liu H., Seynhaeve A.L.B., Brouwer R.W.W., van IJcken W.F., Yang L., Wang Y., Chang Z., ten Hagen T.L. (2019). CREPT Promotes Melanoma Progression Through Accelerated Proliferation and Enhanced Migration by RhoA-Mediated Actin Filaments and Focal Adhesion Formation. Cancers.

[128] Marimuthu M., Rousset N., St-Georges-Robillard A., Lateef M.A., Ferland M., Mes-Masson A.M., Gervais T. (2018). Multi-size spheroid formation using microfluidic funnels. Lab Chip.

[129] Jeong S.Y., Lee J.H., Shin Y., Chung S., Kuh H.J. (2016). Co-Culture of Tumor Spheroids and Fibroblasts in a Collagen Matrix-Incorporated Microfluidic Chip Mimics Reciprocal Activation in Solid Tumor Microenvironment. PLoS ONE.

[130] Feng H., Ou B.C., Zhao J.K., Yin S., Lu A.G., Oechsle E., Thasler W.E. (2017). Homogeneous pancreatic cancer spheroids mimic growth pattern of circulating tumor cell clusters and macrometastases: Displaying heterogeneity and crater-like structure on inner layer. J. Cancer Res. Clin. Oncol..

[131] Ivanov D.P., Parker T.L., Walker D.A., Alexander C., Ashford M.B., Gellert P.R., Garnett M.C. (2014). Multiplexing spheroid volume, resazurin and acid phosphatase viability assays for high-throughput screening of tumour spheroids and stem cell neurospheres. PLoS ONE.

[132] Wang S., Wang X., Boone J., Wie J., Yip K.P., Zhang J., Wang L., Liu R. (2017). Application of Hanging Drop Technique for Kidney Tissue Culture. Kidney Blood Press. Res..

[133] Acar S., Arslan N., Paketçi A., Okur T.D., Demir K., Böber E., Abacı A. (2018). Presentation of central precocious puberty in two patients with Tay-Sachs disease. Hormones.

[134] Jaganathan H., Gage J., Leonard F., Srinivasan S., Souza G.R., Dave B., Godin B. (2014). Three-dimensional in vitro co-culture model of breast tumor using magnetic levitation. Sci. Rep..

[135] Gonzalez-Fernandez T., Tenorio A.J., Leach J.K. (2020). Three-Dimensional Printed Stamps for the Fabrication of Patterned Microwells and High-Throughput Production of Homogeneous Cell Spheroids. 3D Print Addit. Manuf..

[136] Santos J.M., Camões S.P., Filipe E., Cipriano M., Barcia R.N., Filipe M., Teixeira M., Simões S., Gaspar M., Mosqueira D. (2015). Three-dimensional spheroid cell culture of umbilical cord tissue-derived mesenchymal stromal cells leads to enhanced paracrine induction of wound healing. Stem Cell Res. Ther..

[137] Monjaret F., Fernandes M., Duchemin-Pelletier E., Argento A., Degot S., Young J. (2016). Fully Automated One-Step Production of Functional 3D Tumor Spheroids for High-Content Screening. J. Lab. Autom..

[138] Otsuka H., Sasaki K., Okimura S., Nagamura M., Nakasone Y. (2013). Micropatterned co-culture of hepatocyte spheroids layered on non-parenchymal cells to understand heterotypic cellular interactions. Sci. Technol. Adv. Mater..

[139] Abe Y., Tada A., Isoyama J., Nagayama S., Yao R., Adachi J., Tomonaga T. (2018). Improved phosphoproteomic analysis for phosphosignaling and active-kinome profiling in Matrigel-embedded spheroids and patient-derived organoids. Sci. Rep..

[140] Friedrich J., Seidel C., Ebner R., Kunz-Schughart L.A. (2009). Spheroid-based drug screen: Considerations and practical approach. Nat. Protoc..

[141] Zanoni M., Pignatta S., Arienti C., Bonafè M., Tesei A. (2019). Anticancer drug discovery using multicellular tumor spheroid models. Expert Opin. Drug Discov..

[142] Zanoni M., Piccinini F., Arienti C., Zamagni A., Santi S., Polico R., Bevilacqua A., Tesei A. (2016). 3D tumor spheroid models for in vitro therapeutic screening: A systematic approach to enhance the biological relevance of data obtained. Sci. Rep..

[143] Piccinini F., Tesei A., Arienti C., Bevilacqua A. (2015). Cancer multicellular spheroids: Volume assessment from a single 2D projection. Comput. Methods Programs Biomed..

[144] Correa de Sampaio P., Auslaender D., Krubasik D., Failla A.V., Skepper J.N., Murphy G., English W.R. (2012). A heterogeneous in vitro three dimensional model of tumour-stroma interactions regulating sprouting angiogenesis. PLoS ONE.

[145] Ingeson-Carlsson C., Martinez-Monleon A., Nilsson M. (2015). Differential effects of MAPK pathway inhibitors on migration and invasiveness of BRAF(V600E) mutant thyroid cancer cells in 2D and 3D culture. Exp. Cell Res..

[146] Moraes G.S., Wink M.R., Klamt F., Silva A.O., da Cruz Fernandes M. (2020). Simplified low-cost methodology to establish, histologically process and analyze three-dimensional cancer cell spheroid arrays. Eur. J. Cell Biol..

[147] Huisken J., Swoger J., Del Bene FWittbrodt J., Stelzer E.H. (2004). Optical sectioning deep inside live embryos by selective plane illumination microscopy. Science.

[148] Cella Zanacchi F., Lavagnino Z., Perrone Donnorso M., Del Bue A., Furia L., Faretta M., Diaspro A. (2011). Live-cell 3D super-resolution imaging in thick biological samples. Nat. Methods.

[149] Hwang Y.J., Kolettis N., Yang M., Gillard E.R., Sanchez E., Sun C.H., Tromberg B.J., Krasieva T.B., Lyubovitsky J.G. (2011). Multiphoton imaging of actin filament formation and mitochondrial energetics of human ACBT gliomas. Photochem. Photobiol..

[150] Tesei A., Sarnelli A., Arienti C., Menghi E., Medri L., Gabucci E., Pignatta S., Falconi M., Silvestrini R., Zoli W. (2013). In vitro irradiation system for radiobiological experiments. Radiat. Oncol..

[151] Huang K., Ma H., Liu J., Huo S., Kumar A., Wei T., Zhang X., Jin S., Gan Y., Wang P.C. (2012). Size-dependent localization and penetration of ultrasmall gold nanoparticles in cancer cells, multicellular spheroids, and tumors in vivo. ACS Nano.

[152] Costa E.C., Gaspar V.M., Coutinho P., Correia I.J. (2014). Optimization of liquid overlay technique to formulate heterogenic 3D co-cultures models. Biotechnol. Bioeng..

[153] Yao H.J., Ju R.J., Wang X., Zhang Y., Li R.J., Yu Y., Zhang L., Lu W.L. (2011). The antitumor efficacy of functional paclitaxel nanomicelles in treating resistant breast cancers by oral delivery. Biomaterials.

[154] Uroukov I.S., Patton D. (2008). Optimizing environmental scanning electron microscopy of spheroidal reaggregated neuronal cultures. Microsc. Res. Tech..

[155] Patra B., Peng C.C., Liao W.H., Lee C.H., Tung Y.C. (2016). Drug testing and flow cytometry analysis on a large number of uniform sized tumor spheroids using a microfluidic device. Sci. Rep..

[156] Beaumont K.A., Anfosso A., Ahmed F., Weninger W., Haass N.K. (2015). Imaging- and Flow Cytometry-based Analysis of Cell Position and the Cell Cycle in 3D Melanoma Spheroids. J. Vis. Exp..

[157] Durand R.E. (1982). Use of Hoechst 33342 for cell selection from multicell systems. J. Histochem. Cytochem..

[158] Ho W.Y., Yeap S.K., Ho C.L., Rahim R.A., Alitheen N.B. (2012). Development of multicellular tumor spheroid (MCTS) culture from breast cancer cell and a high throughput screening method using the MTT assay. PLoS ONE.

[159] Solomon M.A., Lemera J., D’Souza G.G. (2016). Development of an in vitro tumor spheroid culture model amenable to high-throughput testing of potential anticancer nanotherapeutics. J. Liposome Res..

[160] Kessel S., Cribbes S., Bonasu S., Rice W., Qiu J., Chan L.L.Y. (2017). Real-time viability and apoptosis kinetic detection method of 3D multicellular tumor spheroids using the Celigo Image Cytometer. Cytom. A.

[161] Pignatta S., Orienti I., Falconi M., Teti G., Arienti C., Medri L., Zanoni M., Carloni S., Zoli W., Amadori D. (2015). Albumin nanocapsules containing fenretinide: Pre-clinical evaluation of cytotoxic activity in experimental models of human non-small cell lung cancer. Nanomedicine.

[162] Xin H., Sha X., Jiang X., Zhang W., Chen L., Fang X. (2012). Anti-glioblastoma efficacy and safety of paclitaxel-loading Angiopep-conjugated dual targeting PEG-PCL nanoparticles. Biomaterials.

[163] Robertson F., Ogasawara M.A., Ye Z., Chu K., Pickei R., Debeb B.G., Woodward W.A., Hittelman W.N., Cristofanilli M., Barsky S.H. (2010). Imaging and analysis of 3D tumor spheroids enriched for a cancer stem cell phenotype. J. Biomol. Screen.

[164] Schneckenburger H., Weber P., Wagner M., Schickinger S., Richter V., Bruns T., Strauss W., Wittig R. (2011). Light exposure and cell viability in fluorescence microscopy. J. Microsc..

[165] Darrigues E., Nima Z.A., Nedosekin D.A., Watanabe F., Alghazali K.M., Zharov V.P., Biris A.S. (2020). Tracking Gold Nanorods’ Interaction with Large 3D Pancreatic-Stromal Tumor Spheroids by Multimodal Imaging, Fluorescence, Photoacoustic, and Photothermal Microscopies. Sci. Rep..

[166] Vinci M., Gowan S., Boxall F., Patterson L., Zimmermann M., Court W., Lomas C., Mendiola M., Hardisson D., Eccles S.A. (2012). Advances in establishment and analysis of three-dimensional tumor spheroid-based functional assays for target validation and drug evaluation. BMC Biol..

[167] Yakavets I., Jenard S., Francois A., Maklygina Y., Loschenov V., Lassalle H.-P., Dolivet G., Bezdetnaya L. (2019). Stroma-Rich Co-Culture Multicellular Tumor Spheroids as a Tool for Photoactive Drugs Screening. J. Clin. Med..

[168] Shi W., Kwon J., Huang Y., Tan J., Uhl C.G., He R., Zhou C., Liu Y. (2018). Facile Tumor Spheroids Formation in Large Quantity with Controllable Size and High Uniformity. Sci. Rep..

[169] Ivanov D.P., Grabowska A.M. (2017). Spheroid arrays for high-throughput single-cell analysis of spatial patterns and biomarker expression in 3D. Sci. Rep..

[170] Ansari N., Müller S., Stelzer E.H., Pampaloni F. (2013). Quantitative 3D cell-based assay performed with cellular spheroids and fluorescence microscopy. Methods Cell Biol..

[171] Leary E., Rhee C., Wilks B.T., Morgan J.R. (2018). Quantitative Live-Cell Confocal Imaging of 3D Spheroids in a High-Throughput Format. SLAS Technol..

[172] Durymanov M., Kroll C., Permyakova A., O’Neill E., Sulaiman R., Person M., Reineke J. (2019). Subcutaneous Inoculation of 3D Pancreatic Cancer Spheroids Results in Development of Reproducible Stroma-Rich Tumors. Transl. Oncol..

[173] Nürnberg E., Vitacolonna M., Klicks J., Von Molitor E., Cesetti T., Keller F., Bruch R., Ertongur-Fauth T., Riedel K., Scholz P. (2020). Routine Optical Clearing of 3D-Cell Cultures, Simplicity Forward. Front. Mol. Biosci..

[174] Diaspro A., Federici F., Robello M. (2002). Influence of refractive-index mismatch in high-resolution three-dimensional confocal microscopy. Appl. Opt..

[175] Lazzari G., Vinciguerra D., Balasso A., Nicolas V., Goudin N., Garfa-Traore M., Féher A., Dinnyés A., Nicolas J., Couvreur P. (2019). Light sheet fluorescence microscopy versus confocal microscopy: In quest of a suitable tool to assess drug and nanomedicine penetration into multicellular tumor spheroids. Eur. J. Pharm. Biopharm..

[176] Buglak N.E., Lucitti J., Ariel P., Maiocchi S.L., Miller F.J., Bahnson E.S.M. (2020). Light Sheet Fluorescence Microscopy as a New Method for Unbiased Three-Dimensional Analysis of Vascular Injury. Cardiovasc. Res.

[177] Smyrek I., Stelzer E.H. (2017). Quantitative three-dimensional evaluation of immunofluorescence staining for large whole mount spheroids with light sheet microscopy. Biomed. Opt. Express.

[178] Lazzari G., Nicolas V., Matsusaki M., Akashi M., Couvreur P., Mura S. (2018). Multicellular spheroid based on a triple co-culture, A novel 3D model to mimic pancreatic tumor complexity. Acta Biomater..

[179] Stern T., Kaner I., Zer N.L., Shoval H., Dror D., Manevitch Z., Chai L., Brill-Karniely Y., Benny O. (2017). Rigidity of polymer micelles affects interactions with tumor cells. J. Control. Release.

[180] Wan X., Li Z., Ye H., Cui Z. (2016). Three-dimensional perfused tumour spheroid model for anti-cancer drug screening. Biotechnol. Lett..

[181] Nylk J., McCluskey K., Preciado M.A., Mazilu M., Yang Z., Gunn-Moore F.J., Aggarwal S., Tello J.A., Ferrier D.E.K., Dholakia K. (2018). Light-sheet microscopy with attenuation-compensated propagation-invariant beams. Sci. Adv..

[182] Shemesh Z., Chaimovich G., Gino L., Ozana N., Nylk J., Dholakia K., Zalevsky Z. (2020). Reducing data acquisition for light-sheet microscopy by extrapolation between imaged planes. J. Biophotonics.

[183] Zhao F., Yang Y., Li Y., Jiang H., Xie X., Yu T., Wang X., Liu Q., Zhang H., Jia H. (2020). Efficient and cost-effective 3D cellular imaging by sub-voxel-resolving light-sheet add-on microscopy. J. Biophotonics.

[184] Lin H., Fan T., Sui J., Wang G., Chen J., Zhuo S., Zhang H. (2019). Recent advances in multiphoton microscopy combined with nanomaterials in the field of disease evolution and clinical applications to liver cancer. Nanoscale.

[185] Sato R., Yasukawa T., Kacza J., Eichler W., Nishiwaki A., Iandiev I., Ohbayashi M., Kato A., Yafai Y., Bringmann A. (2013). Three-Dimensional Spheroidal Culture Visualization of Membranogenesis of Bruch’s Membrane and Basolateral Functions of the Retinal Pigment Epithelium. Investig. Ophthalmol. Vis. Sci..

[186] Beauchamp P., Jackson C.B., Ozhathil L.C., Agarkova I., Galindo C.L., Sawyer D.B., Suter T.M., Zuppinger C. (2020). 3D Co-culture of hiPSC-Derived Cardiomyocytes With Cardiac Fibroblasts Improves Tissue-Like Features of Cardiac Spheroids. Front. Mol. Biosci..

[187] Hortelão A.C., Carrascosa R., Murillo-Cremaes N., Patiño T., Sanchez S. (2019). Targeting 3D Bladder Cancer Spheroids with Urease-Powered Nanomotors. ACS Nano.

[188] Chelobanov B., Poletaeva J., Epanchintseva A., Tupitsyna A., Pyshnaya I., Ryabchikova E. (2020). Ultrastructural Features of Gold Nanoparticles Interaction with HepG2 and HEK293 Cells in Monolayer and Spheroids. Nanomaterials.

[189] Salehi F., Behboudi H., Kavoosi G., Ardestani S.K. (2017). Monitoring ZEO apoptotic potential in 2D and 3D cell cultures and associated spectroscopic evidence on mode of interaction with DNA. Sci. Rep..

[190] Nigjeh S.E., Yeap S.K., Nordin N., Kamalideghan B., Ky H., Rosli R. (2018). Citral induced apoptosis in MDA-MB-231 spheroid cells. BMC Complement. Altern. Med..

[191] Salehi F., Jamali T., Kavoosi G., Ardestani S.K., Vahdati S.N. (2020). Stabilization of Zataria essential oil with pectin-based nanoemulsion for enhanced cytotoxicity in monolayer and spheroid drug-resistant breast cancer cell cultures and deciphering its binding mode with gDNA. Int. J. Biol. Macromol..

[192] Mirab F., Kang Y.J., Majd S. (2019). Preparation and characterization of size-controlled glioma spheroids using agarose hydrogel microwells. PLoS ONE.

[193] Svirshchevskaya E., Doronina E., Grechikhina M., Matushevskaya E., Kotsareva O., Fattakhova G., Sapozhnikov A., Felix K. (2019). Characteristics of multicellular tumor spheroids formed by pancreatic cells expressing different adhesion molecules. Life Sci..

[194] Khaitan D., Chandna S., Arya M.B., Dwarakanath B.S. (2006). Establishment and characterization of multicellular spheroids from a human glioma cell line; Implications for tumor therapy. J. Transl. Med..

[195] Liu J., Li J., Li P., Wang Y., Liang Z., Jiang Y., Li J., Feng C., Wang R., Chen H. (2017). Loss of DLG5 promotes breast cancer malignancy by inhibiting the Hippo signaling pathway. Sci. Rep..

[196] Wang Q., Bu S., Xin D., Li B., Wang L., Lai D. (2018). Autophagy Is Indispensable for the Self-Renewal and Quiescence of Ovarian Cancer Spheroid Cells with Stem Cell-Like Properties. Oxidative Med. Cell. Longev..

[197] Bauleth-Ramos T., Feijão T., Gonçalves A., Shahbazi M.-A., Liu Z., Barrias C., Oliveira M.J., Granja P., Santos H.A., Sarmento B. (2020). Colorectal cancer triple co-culture spheroid model to assess the biocompatibility and anticancer properties of polymeric nanoparticles. J. Control. Release.

[198] Guo X., Chen Y., Ji W., Chen X., Li C., Ge R. (2019). Enrichment of cancer stem cells by agarose multi-well dishes and 3D spheroid culture. Cell Tissue Res..

[199] Lu H., Stenzel M.H. (2018). Multicellular Tumor Spheroids (MCTS) as a 3D In Vitro Evaluation Tool of Nanoparticles. Small.

[200] Askari E., Naghib S.M., Seyfoori A., Maleki A., Rahmanian M. (2019). Ultrasonic-assisted synthesis and in vitro biological assessments of a novel herceptin-stabilized graphene using three dimensional cell spheroid. Ultrason Sonochemistry.

[201] Flampouri E., Imar S., Oconnell K., Singh B. (2019). Spheroid-3D and Monolayer-2D Intestinal Electrochemical Biosensor for Toxicity/Viability Testing, Applications in Drug Screening, Food Safety, and Environmental Pollutant Analysis. ACS Sens..

[202] Friedrich J., Eder W., Castaneda J., Doss M., Huber E., Ebner R., Kunz-Schughart L.A. (2007). A reliable tool to determine cell viability in complex 3-d culture: The acid phosphatase assay. J. Biomol. Screen..

[203] Rolver M.G., Elingaard-Larsen L.O., Pedersen S.F. (2019). Assessing Cell Viability and Death in 3D Spheroid Cultures of Cancer Cells. J. Vis. Exp..

[204] Kijanska M., Kelm J., Markossian S., Sittampalam G.S., Grossman A., Brimacombe K., Arkin M., Auld D., Austin C.P., Baell J., Caaveiro J.M.M., Chung T.D.Y. (2004). In vitro 3D Spheroids and Microtissues, ATP-based Cell Viability and Toxicity Assays. Assay Guidance Manual.

[205] Aughton K., Shahidipour H., Djirackor L., Coupland S.E., Kalirai H. (2020). Characterization of Uveal Melanoma Cell Lines and Primary Tumor Samples in 3D Culture. Transl. Vis. Sci. Technol..

[206] Huang Z., Yu P., Tang J. (2020). Characterization of Triple-Negative Breast Cancer MDA-MB-231 Cell Spheroid Model. OncoTargets Ther..

[207] Posimo J.M., Unnithan A.S., Gleixner A.M., Choi H.J., Jiang Y., Pulugulla S.H., Leak R.K. (2014). Viability assays for cells in culture. J. Vis. Exp..

[208] Norberg K.J., Liu X., Moro C.F., Strell C., Nania S., Blümel M., Balboni A., Bozóky B., Heuchel R.L., Löhr J.M. (2020). A novel pancreatic tumour and stellate cell 3D co-culture spheroid model. BMC Cancer.

[209] Sano K., Usui M., Moritani Y., Nakazawa K., Hanatani T., Kondo H., Nakatomi M., Onizuka S., Iwata T., Sato T. (2020). Co-cultured spheroids of human periodontal ligament mesenchymal stem cells and vascular endothelial cells enhance periodontal tissue regeneration. Regen. Ther..

[210] Mori Y., Yamawaki K., Ishiguro T., Yoshihara K., Ueda H., Sato A., Ohata H., Yoshida Y., Minamino T., Okamoto K. (2019). ALDH-Dependent Glycolytic Activation Mediates Stemness and Paclitaxel Resistance in Patient-Derived Spheroid Models of Uterine Endometrial Cancer. Stem Cell Rep..

[211] Joshi J., Mahajan G., Kothapalli C.R. (2018). Three-dimensional collagenous niche and azacytidine selectively promote time-dependent cardiomyogenesis from human bone marrow-derived MSC spheroids. Biotechnol. Bioeng..

[212] Khaitan D., Dwarakanath B.S. (2006). Multicellular spheroids as an in vitro model in experimental oncology: Applications in translational medicine. Expert Opin. Drug Discov..

[213] Sacks D., Baxter B., Campbell B.C., Carpenter J.S., Cognard C., Dippel D., Eesa M., Fischer U., Hausegger K., Hirsch J.A. (2018). Multisociety Consensus Quality Improvement Revised Consensus Statement for Endovascular Therapy of Acute Ischemic Stroke. Int. J. Stroke.

[214] Barbone D., Yang T.M., Morgan J., Gaudino G., Broaddus V.C. (2008). Mammalian target of rapamycin contributes to the acquired apoptotic resistance of human mesothelioma multicellular spheroids. J. Biol. Chem..

[215] Huanwen W., Zhiyong L., Xiaohua S., Xinyu R., Kai W., Tonghua L. (2009). Intrinsic chemoresistance to gemcitabine is associated with constitutive and laminin-induced phosphorylation of FAK in pancreatic cancer cell lines. Mol. Cancer.

[216] Thomas F., Holly J.M., Persad R., Bahl A., Perks C.M. (2010). Fibronectin confers survival against chemotherapeutic agents but not against radiotherapy in DU145 prostate cancer cells: Involvement of the insulin like growth factor-1 receptor. Prostate.

[217] Weigelt B., Lo A.T., Park C.C., Gray J.W., Bissell M.J. (2010). HER2 signaling pathway activation and response of breast cancer cells to HER2-targeting agents is dependent strongly on the 3D microenvironment. Breast Cancer Res. Treat..

[218] Liao J., Qian F., Tchabo N., Mhawech-Fauceglia P., Beck A., Qian Z., Wang X., Huss W.J., Lele S.B., Morrison C.D. (2014). Ovarian cancer spheroid cells with stem cell-like properties contribute to tumor generation, metastasis and chemotherapy resistance through hypoxia-resistant metabolism. PLoS ONE.

[219] Wartenberg M., Hoffmann E., Schwindt H., Grünheck F., Petros J., Arnold J.R.S., Hescheler J., Sauer H. (2005). Reactive oxygen species-linked regulation of the multidrug resistance transporter P-glycoprotein in Nox-1 overexpressing prostate tumor spheroids. FEBS Lett..

[220] Hoffmann O., Ilmberger C., Magosch S., Joka M., Jauch K.-W., Mayer B. (2015). Impact of the spheroid model complexity on drug response. J. Biotechnol..

[221] Sethi T., Rintoul R.C., Moore S.M., MacKinnon A.C., Salter D., Choo C., Chilvers E.R., Dransfield I., Donnelly S.C., Strieter R.M. (1999). Extracellular matrix proteins protect small cell lung cancer cells against apoptosis: A mechanism for small cell lung cancer growth and drug resistance in vivo. Nat. Med..

[222] Aoudjit F., Vuori K. (2001). Integrin signaling inhibits paclitaxel-induced apoptosis in breast cancer cells. Oncogene.

[223] Nunes A.S., Barros A.S., Costa E.C., Moreira A.F., Correia I.J. (2019). 3D tumor spheroids as in vitro models to mimic in vivo human solid tumors resistance to therapeutic drugs. Biotechnol. Bioeng..

[224] Frankel A., Man S., Elliott P., Adams J., Kerbel R.S. (2000). Lack of multicellular drug resistance observed in human ovarian and prostate carcinoma treated with the proteasome inhibitor PS-341. Clin. Cancer Res..

[225] Ferrante A., Rainaldi G., Indovina P., Indovina P.L., Santini M.T. (2006). Increased cell compaction can augment the resistance of HT-29 human colon adenocarcinoma spheroids to ionizing radiation. Int. J. Oncol..

[226] Olive P.L., Durand R.E. (1994). Drug and radiation resistance in spheroids: Cell contact and kinetics. Cancer Metastasis Rev..

[227] Robert Grimes D., Partridge M. (2015). A mechanistic investigation of the oxygen fixation hypothesis and oxygen enhancement ratio. Biomed. Phys. Eng. Express.

[228] Horan A.D., Giandomenico A.R., Koch C.J. (1999). Effect of oxygen on radiation-induced DNA damage in isolated nuclei. Radiat. Res..

[229] Lefranc F., Brotchi J., Kiss R. (2005). Possible future issues in the treatment of glioblastomas: Special emphasis on cell migration and the resistance of migrating glioblastoma cells to apoptosis. J. Clin. Oncol..

[230] Erler J.T., Weaver V.M. (2009). Three-dimensional context regulation of metastasis. Clin. Exp. Metastasis.

[231] Jessup J.M., Brown D., Fitzgerald W., Ford R.D., Nachman A., Goodwin T.J., Spaulding G. (1997). Induction of carcinoembryonic antigen expression in a three-dimensional culture system. In Vitro Cell Dev. Biol. Anim..

[232] Vinci M., Box C., Zimmermann M., Eccles S.A. (2013). Tumor spheroid-based migration assays for evaluation of therapeutic agents. Methods Mol. Biol..

[233] Weiswald L.B., Bellet D., Dangles-Marie V. (2015). Spherical cancer models in tumor biology. Neoplasia.

[234] Cattin S., Ramont L., Rüegg C. (2018). Characterization and In Vivo Validation of a Three-Dimensional Multi-Cellular Culture Model to Study Heterotypic Interactions in Colorectal Cancer Cell Growth, Invasion and Metastasis. Front. Bioeng. Biotechnol..

[235] Almahmoudi R., Salem A., Murshid S., Dourado M.R., Apu E.H., Salo T., Al-Samadi A. (2019). Interleukin-17F Has Anti-Tumor Effects in Oral Tongue Cancer. Cancers.

[236] Gao Q., Yang Z., Xu S., Li X., Yang X., Jin P., Liu Y., Zhou X., Zhang T., Gong C. (2019). Heterotypic CAF-tumor spheroids promote early peritoneal metastatis of ovarian cancer. J. Exp. Med..

[237] Yamamoto S., Hotta M.M., Okochi M., Honda H. (2014). Effect of vascular formed endothelial cell network on the invasive capacity of melanoma using the in vitro 3D co-culture patterning model. PLoS ONE.

[238] Vinci M., Box C., Eccles S.A. (2015). Three-dimensional (3D) tumor spheroid invasion assay. J. Vis. Exp..

[239] Berens E.B., Holy J.M., Riegel A.T., Wellstein A. (2015). A Cancer Cell Spheroid Assay to Assess Invasion in a 3D Setting. J. Vis. Exp..

[240] De Wever O., Hendrix A., De Boeck A., Eertmans F., Westbroek W., Braems G., Bracke M.E. (2014). Single cell and spheroid collagen type I invasion assay. Methods Mol. Biol..

[241] Febles N.K., Ferrie A.M., Fang Y. (2014). Label-free single cell kinetics of the invasion of spheroidal colon cancer cells through 3D Matrigel. Anal. Chem..

[242] Bell H.S., Wharton S.B., Leaver H.A., Whittle I.R. (1999). Effects of N-6 essential fatty acids on glioma invasion and growth: Experimental studies with glioma spheroids in collagen gels. J. Neurosurg..

[243] Marchal S., El Hor A., Millard M., Gillon V., Bezdetnaya L. (2015). Anticancer Drug Delivery, An Update on Clinically Applied Nanotherapeutics. Drugs.

[244] Aldawsari H.M., Singh S. (2020). Rapid Microwave-Assisted Cisplatin-Loaded Solid Lipid Nanoparticles, Synthesis, Characterization and Anticancer Study. Nanomaterials.

[245] Wang H., Li L., Ye J., Wang R., Wang R., Hu J., Wang Y., Dong W., Xia X., Yang Y. (2020). Improving the Oral Bioavailability of an Anti-Glioma Prodrug CAT3 Using Novel Solid Lipid Nanoparticles Containing Oleic Acid-CAT3 Conjugates. Pharmaceutics.

[246] Zielińska A., Ferreira N.R., Durazzo A., Lucarini M., Cicero N., Mamouni S.E., Silva A.M., Nowak I., Santini A., Souto E.B. (2019). Development and Optimization of Alpha-Pinene-Loaded Solid Lipid Nanoparticles (SLN) Using Experimental Factorial Design and Dispersion Analysis. Molecules.

[247] Lukowski J.K., Hummon A.B. (2019). Quantitative evaluation of liposomal doxorubicin and its metabolites in spheroids. Anal. Bioanal. Chem..

[248] Yang S., Gao H. (2017). Nanoparticles for modulating tumor microenvironment to improve drug delivery and tumor therapy. Pharmacol. Res..

[249] Davies Cde L., Berk D.A., Pluen A., Jain R.K. (2002). Comparison of IgG diffusion and extracellular matrix composition in rhabdomyosarcomas grown in mice versus in vitro as spheroids reveals the role of host stromal cells. Br. J. Cancer.

[250] Hobbs S.K., Monsky W.L., Yuan F., Roberts W.G., Griffith L., Torchilin V.P., Jain R.K. (1998). Regulation of transport pathways in tumor vessels: Role of tumor type and microenvironment. Proc Natl Acad Sci USA.

[251] Prabhakar U., Maeda H., Jain R.K., Sevick-Muraca E.M., Zamboni W., Farokhzad O.C., Barry S.T., Gabizon A., Grodzinski P., Blakey D.C. (2013). Challenges and key considerations of the enhanced permeability and retention effect for nanomedicine drug delivery in oncology. Cancer Res..

[252] Patel N.R., Aryasomayajula B., Abouzeid A.H., Torchilin V.P. (2015). Cancer cell spheroids for screening of chemotherapeutics and drug-delivery systems. Ther. Deliv..

[253] Jin H., Gui R., Sun J., Wang Y. (2018). RETRACTED, Facilely self-assembled magnetic nanoparticles/aptamer/carbon dots nanocomposites for highly sensitive up-conversion fluorescence turn-on detection of tetrodotoxin. Talanta.

[254] Millard M., Yakavets I., Zorin V., Kulmukhamedova A., Marchal S., Bezdetnaya L. (2017). Drug delivery to solid tumors: The predictive value of the multicellular tumor spheroid model for nanomedicine screening. Int. J. Nanomed..

[255] Bugno J., Hsu H.-J., Pearson R.M., Noh H., Hong S. (2016). Size and Surface Charge of Engineered Poly(amidoamine) Dendrimers Modulate Tumor Accumulation and Penetration, A Model Study Using Multicellular Tumor Spheroids. Mol. Pharm..

[256] Ni D., Ding H., Liu S., Yue H., Bao Y., Wang Z., Su Z., Wei W., Ma G. (2015). Superior intratumoral penetration of paclitaxel nanodots strengthens tumor restriction and metastasis prevention. Small.

[257] Agarwal R., Jurney P., Raythatha M., Singh V., Sreenivasan S.V., Shi L., Roy K. (2015). Effect of shape, size, and aspect ratio on nanoparticle penetration and distribution inside solid tissues using 3D spheroid models. Adv. Healthc. Mater..

[258] Goodman T.T., Olive P.L., Pun S.H. (2007). Increased nanoparticle penetration in collagenase-treated multicellular spheroids. Int. J. Nanomed..

[259] Hinger D., Navarro F.P., Käch A., Thomann J.-S., Mittler F., Couffin A.-C., Maake C. (2016). Photoinduced effects of m-tetrahydroxyphenylchlorin loaded lipid nanoemulsions on multicellular tumor spheroids. J. Nanobiotechnology.

[260] Ernsting M.J., Murakami M., Roy A., Li S.D. (2013). Factors controlling the pharmacokinetics, biodistribution and intratumoral penetration of nanoparticles. J Control. Release.

[261] Kim B., Han G., Toley B.J., Kim C., Rotello V.M., Forbes N.S. (2010). Tuning payload delivery in tumour cylindroids using gold nanoparticles. Nat. Nanotechnol..

[262] Chauhan V.P., Popović Z., Chen O., Cui J., Fukumura D., Bawendi M.G., Jain R.K. (2011). Fluorescent nanorods and nanospheres for real-time in vivo probing of nanoparticle shape-dependent tumor penetration. Angew. Chem. Int. Ed. Engl..

[263] Wang Y., Kibbe M.R., Ameer G.A. (2013). Photo-crosslinked Biodegradable Elastomers for Controlled Nitric Oxide Delivery. Biomater. Sci..

[264] Zhao J., Lu H., Xiao P., Stenzel M.H. (2016). Cellular Uptake and Movement in 2D and 3D Multicellular Breast Cancer Models of Fructose-Based Cylindrical Micelles That Is Dependent on the Rod Length. ACS Appl. Mater. Interfaces.

[265] Michy T., Massias T., Bernard C., VanWonterghem L., Henry M., Guidetti M., Royal G., Coll J.-L., Texier I., Josserand V. (2019). Verteporfin-Loaded Lipid Nanoparticles Improve Ovarian Cancer Photodynamic Therapy In Vitro and In Vivo. Cancers.

[266] Chen H., Wei X., Chen H., Wei H., Wang Y., Nan W., Zhang Q., Wen X. (2019). The study of establishment of an in vivo tumor model by three-dimensional cells culture systems methods and evaluation of antitumor effect of biotin-conjugated pullulan acetate nanoparticles. Artif. Cells Nanomed. Biotechnol..

[267] Wang X., Zhen X., Wang J., Zhang J., Wu W., Jiang X. (2013). Doxorubicin delivery to 3D multicellular spheroids and tumors based on boronic acid-rich chitosan nanoparticles. Biomaterials.

